# Morphine- and foot shock-responsive neuronal ensembles in the VTA possess different connectivity and biased GPCR signaling pathway

**DOI:** 10.7150/thno.90792

**Published:** 2024-01-12

**Authors:** Fan Wang, Chao-bao Liu, Yi Wang, Xi-xi Wang, Yuan-yao Yang, Chang-you Jiang, Qiu-min Le, Xing Liu, Lan Ma, Fei-fei Wang

**Affiliations:** 1 School of Basic Medical Sciences, MOE Frontiers Center for Brain Science, Institutes of Brain Science, State Key Laboratory of Medical Neurobiology, Pharmacology Research Center, Department of Neurology, Huashan Hospital, Fudan University, Shanghai 200032, China.; 2 Research Unit of Addiction Memory, Chinese Academy of Medical Sciences (2021RU009), Shanghai 200032, China.

**Keywords:** Ventral tegmental area, Morphine, Foot shock, Ensemble, GPCR

## Abstract

**Background:** Neurons in the ventral tegmental area (VTA) are sensitive to stress and their maladaptation have been implicated in the psychiatric disorders such as anxiety and addiction,* etc*. The cellular properties of the VTA neurons in response to different stressors related to different emotional processing remain to be investigated.

**Methods:** By combining immediate early gene (IEG)-dependent labeling, rabies virus tracing, ensemble-specific transcriptomic analysis and fiber photometry recording in the VTA of male mice, the spatial distribution, brain-wide connectivity and cellular signaling pathways in the VTA neuronal ensembles in response to morphine (Mor-Ens) or foot shock (Shock-Ens) stimuli were investigated.

**Results:** Optogenetic activation of the Mor-Ens drove approach behavior, whereas chemogenetic activation of the Shock-Ens increased the anxiety level in mice. Mor-Ens were clustered and enriched in the ventral VTA, contained a higher proportion of dopaminergic neurons, received more inputs from the dorsal medial striatum and the medial hypothalamic zone, and exhibited greater axonal arborization in the zona incerta and ventral pallidum. Whereas Shock-Ens were more dispersed, contained a higher proportion of GABAergic neurons, and received more inputs from the ventral pallidum and the lateral hypothalamic area. The downstream targets of the G protein and β-arrestin pathways, PLCβ3 and phosphorylated AKT1^Thr308^, were relatively enriched in the Mor-Ens and Shock-Ens, respectively. Cariprazine, the G-protein-biased agonist for the dopamine D2 receptor, increased the response of Mor-Ens to sucrose water and decreased the anxiety-like behavior during morphine withdrawal, whereas the β-arrestin-biased agonist UNC9994 decreased the response of Shock-Ens to tail suspension.

**Conclusions:** Taken together, these findings reveal the heterogeneous connectivity and signaling pathways of the VTA neurons in response to morphine and foot shock, providing new insights for development of specific interventions for psychiatric disorders caused by various stressors associated with different VTA neuronal functions.

## Introduction

Maladaptive and uncontrollable responses to environmental stressors have been linked to psychiatric disorders, such as anxiety, post-traumatic stress disorder (PTSD) and addiction, *etc.* 1-5. Studies in rodent models have revealed multiple mechanisms related to the ventral tegmental area (VTA), a midbrain area that plays a significant role in emotional processing in stress or psychoactive substance-induced pathological changes 6-8. VTA neurons are heterogeneous in their afferent and efferent connectivity. Various inputs, outputs, and local connections have been shown to contribute to the generation of reward- or aversion-related behaviors 6, 7. The diverse organization of connectivity and neuronal ensembles within the VTA are thought to be responsible for mediating complex behaviors by integrating stressful or pathological stimuli to produce specific behavioral outcomes 7.

Previous studies have shown that neurons in different subregions of the VTA are connected to different neural circuits and play different roles in reinforcement, motivation and learning. The activity of glutamate inputs to VTA glutamatergic neurons increases for both reward-predicting and aversive-predicting cues, whereas the activity of GABAergic inputs to VTA glutamatergic neurons mostly decreases for both reward and aversive cues and outcomes 8. Monosynaptic tracing studies reveal that dopaminergic neurons in different spatial locations of the VTA have segregated outputs and distinct input patterns 9. VTA dopaminergic (DA) neurons that project to the nucleus accumbens (NAc) rather than to the amygdala nuclei mediate anxiety-like behavior 10, 11. The outputs of VTA neurons are integrated not only with inputs from other brain regions but also with inputs from local GABAergic and glutamatergic micro-circuits 7. GABAergic neurons in the rostral VTA innervate GABAergic neurons in the dorsal raphe nucleus (DRN) and mediate aversion, while GABAergic neurons in the caudal VTA primarily innervate 5-HTergic neurons in the DRN and mediate reward 12. Foot shock of varying intensities produce behavioral and neurochemical changes that reflect depression, anxiety, and PTSD in humans and animal models 13, 14. While administration of opioids, a widely used analgesic drug, induce euphoria 15. A single intraperitoneal (i.p.) injection of morphine results in a preference for the injection side 16 and activating VTA neuronal ensembles which induces dopamine dependent positive reinforcement 17, indicating the rewarding effect of morphine. Recent studies have revealed the heterogeneity of neuronal ensembles and their differential responses to various emotional stimuli. Studies using TAI-FISH revealed that neural ensembles responding to appetitive and aversive stimuli have distinct spatial patterns (interaction, segregation, convergence and intermingled) in different brain regions 16. Neuronal ensembles in the ventral hippocampus labelled by activity-dependent strategies responding to appetitive and aversive stimuli are partially segregated and have distinct projection patterns 18. Analysis of neuronal subclusters by single-nucleus RNA sequencing from the rat VTA identifies selective markers for dopamine and combinatorial neurons, reveals expression profiles for receptors targeted by drugs of abuse, and demonstrates population-specific enrichment of gene sets linked to brain disorders 19. However, how circuit connectivity and molecular properties of the VTA neurons cooperatively regulate emotional processing in response to different stressors remain to be investigated.

Noxious and anxiogenic stimuli modulate the VTA which is a critical mediator of motivational states. Pain increases the rostromedial tegmental (RMTg) inhibitory input to VTA DA neurons, making them less excitable 20. Morphine not only alleviates pain but also produces euphoria by affecting the activity of VTA DA neurons 21. Aversive conditions such as electric foot shock is multifaceted stressors that involve both physical and emotional elements. They can induce anxiety-like symptoms in rodents 13. By combining immediate early gene (IEG)-dependent labeling with rabies virus tracing, ensemble-specific transcriptomic analysis, and fiber photometry recording in mice VTA, we unraveled the differentially enriched spatial distribution, brain-wide connectivity and cellular signaling pathways in the VTA neuronal ensembles in response to morphine or electric foot shock stimuli. Recognizing the influence of morphine and foot shock, which involve positive and negative emotional states on the VTA, may provide valuable insights into the development of potential interventions novel strategies for psychiatric disorders caused by various stressor related to different emotional processing.

## Materials and Methods

### Animals

Adult C57BL/6 mice were purchased from the Shanghai Laboratory Animal Center (CAS, Shanghai, China). Mice were housed in groups of 4 mice per cage and maintained on a 12 h light/dark cycle (i.e. light on from 8 a.m. to 8 p.m.) with food and water available ad libitum. Male mice aged 8-10 weeks were used in the following experiments. All experiment procedures and operations were strictly in accordance with the National Institutes of Health Guide for the Care and Use of Laboratory and were approved by the Animal Care and Use Committee of the School of Basic Medical Sciences of Fudan University.

### Viral Vectors

The adeno-associated viruses (AAVs) preparations with 2 × 10^12^ infectious units (IU)/mL, and rabies virus (RV) with 2.0 × 10^8^ (IU)/mL were used.* The pAAV-RAM-d2TTA-pA::TRE-Cre-WPRE-pA* plasmid was constructed as previously described 17, 22 and packaged into serotype 9 by OBiO Technology Co., Ltd (Shanghai, China) . *AAV_2/9_-FLEX-NBL10-HA* was purchased from Taitool Bioscience Co., Ltd (Shanghai, China).* AAV_2/9_-CAG-FLEX-jGCaMP7b, AAV_2/9_-EF1α-DIO-hM3Dq-mCherry, AAV_2/9_-EF1α-DIO-mCherry, AAV_2/9_-EF1a-DIO-hChR2(H134R)-EYFP, AAV_2/9_-EF1a-DIO-hChR2(H134R)-mCherry, AAV_2/9_-RAM-d2TTA::TRE-EGFP, AAV_2/9_-EF1a-DIO-H2B-EGFP-T2A-TVA, AAV_2/9_-EF1a-DIO-oRVG and RV-ENVA-△G-dsRed* were purchased from BrainVTA Co., Ltd (Wuhan, China).

### Stereotaxic Surgery

Mice were anesthetized by isoflurane (3.5% for induction, 1.5-2% for maintenance). Microinjections were conducted in the stereotaxic instrument (Stoelting, Kiel, WI, USA) with a 10-μL syringe and a 33-gauge needle (Hamilton, Bonaduz, Switzerland) which were under the control of the UMP3 ultra micro pump (World Precision Instruments, Florida, USA). The viral injection coordinates for VTA were -3.1 mm AP (anteroposterior); ±0.4 mm ML (mediolateral); -4.45 mm DV (dorsoventral) relative to bregma. Each side of the VTA was injected with a 200 nL of AAV at the rates of 50 nL/min. *AAV_2/9_-EF1a-DIO-hChR2(H134R)-EYFP and pAAV-RAM-d2TTA-pA::TRE-Cre-WPRE-pA* were injected unilaterally into the VTA for optogenetics. The needle was remained for at least 5 min after the injection. For the rabies-mediated trans-synaptic tracing, 200 nL of *AAV-RAM-d2TTA::TRE-CRE, AAV-EF1a-DIO-H2B-EGFP-T2A-TVA* and *AAV-EF1a-DIO-oRVG* mixtures was injected into each side of the VTA. One week after the ensembles labeling, *RV-ENVA-△G-dsRed* (150 nL per side) was injected into the same area. For optical stimulation and fiber photometry recording, 200 μm-diameter 0.37 NA optic-fiber cannulas (Inper Tech, Hangzhou, Zhejiang, China) were implanted unilaterally into the VTA (-3.1 mm AP; ±0.35 mm ML; -4.3 mm DV) of mice. Mice were allowed to recover from the surgery at least 2 weeks before subsequent experiments.

### Ensembles Labeling

Before ensembles labeling, mice were gently handled by the experimenter for 5-10 min for 3 days. The doxycline (dox)-dependent tet-off system was used to label the neuronal ensembles. Dox (360304, Huamaike Bio, Beijing, China) containing diet (40 mg/kg) was replaced with standard chow diet 42 h before labeling the ensembles. Mice were received a single intraperitoneal injection of morphine (10 mg/kg) or six 0.75 mA, 2 s foot shocks within 6 min session in the same context. Dox containing diet was replaced with standard chow without any other manipulation for home-cage labeling. Dox-containing diet was reintroduced 6 h after the ensembles labeling.

### Immunofluorescence and Imaging

Mice were transcardially perfused with saline followed by 4% PFA (paraformaldehyde dissolved in PBS) for 6 min. Brains were removed and fixed in 4% PFA overnight and then transferred to 30% sucrose/PBS solution for dehydration. Brains were sliced into 40 μm coronal sections using a Leica CM3050 S Cryostat (Buffalo Grove, IL, USA). For immunofluorescence staining, slices were incubated with primary antibodies in blocking buffer containing 10% goat serum (Jackson ImmunoResearch, USA) and 0.3% Triton X-100 at 4 °C overnight. After being washed in PBS, the slices were incubated with secondary antibody in PBS on a shaker at room temperature for 1.5 h and then stained with DAPI (D9534, Sigma, Germany) for 5 min. Slices were extensively washed in PBS were mounted in Fluor Mount-G mounting medium (0100-01, Southern Biotech, Birmingham, AL, USA). The following primary antibodies were used: Anti-tyrosine hydroxylase (1:200, MAB318, Millipore, USA). Anti-c-Fos (1:1000, ab190289, Abcam, UK). Anti-GABA (1:500, A2052, Sigma-Aldrich, USA). The secondary antibodies used were: Alexa-488 or Cy3 IgG (mouse, rabbit; 1:1,000, Jackson ImmunoResearch, USA). Fluorescent images were captured by a Nikon-A1 confocal microscope (Tokyo, Japan) with the 10 × or 20 × air objective lens.

### RNA Fluorescence in Situ Hybridization

Mice brains were removed after perfusion, and 10-μm coronal sections were sliced from the frozen brain tissue and mounted on Colorfrost Plus microscope slides (Thermo Scientific, Waltham, USA). Slices were pretreated with hydrogen peroxide for 10 min at room temperature, and RNAscope 2.5 Universal Pretreatment Reagents (ACD: 322380, Advanced Cell Diagnostics, Newark, CA, USA) were used to perform target retrieval and proteolysis. In situ hybridization was then performed for 2 h at 40 °C with the probes of *Egfp*-C1 (ACD: 400281-C1) and *Drd2*-C2 (ACD: 406501-C2). After hybridization, RNAscope Multiplex Fluorescent Reagent Kit (ACD: 323110) was used to amplify the signal. After hybridization, the brain section was washed by 1 × wash buffer (PN 310091). Fluorescent images were captured by a Nikon-A1 confocal microscope with the 20 × air objective lens. The mRNA levels of *Drd2* were analyzed by ImageJ (Fiji) (https://imagej.net/software/fiji/).

### Cell Counting

Data were analyzed with ImageJ. The number of RAM-EGFP^+^, TH^+^ and GABA^+^ and their colocalization in the VTA were counted manually by an experimenter who was blinded to the group assignment. For the spatial distribution of neuronal ensembles in the VTA, the *Trainable Weka Segmentation* plugin was used to automatically count the c-Fos^+^ cells in each coronal section of VTA (form -3.0 mm to -3.8 mm AP) and obtain the two-dimensional coordinates for each cell. The area of VTA area was measured by applying scale calibration. The 3D distribution of c-Fos^+^ cells were plotted using the RGL package in R (version 4.2.0). The distances between paired cells were calculated by R using the 2D cell coordinates mentioned above.

For the rabies virus tracing, every fifth 40 μm coronal section was collected across the entire brain. 20 brain regions including the major inputs to the VTA were selected for analysis. Starter cells (EGFP^+^ dsRed^+^ cells in the VTA) were counted manually. The dsRed^+^ cells in other brain regions were automatically counted as mentioned above.

Abbreviations of brain regions: AcbC, accumbens nucleus core; AcbSh medial, accumbens nucleus, medial shell; AcbSh lateral, accumbens nucleus, lateral shell; BLA, Basolateral amygdalar nucleus; BMA, Basomedial amygdalar nucleus; BNST, Bed nuclei of the stria terminalis; DLStr, dorsal lateral striatum; DMStr, dorsal medial striatum; DORpm, Thalamus, polymodal association cortex related; DORsm, Thalamus, sensory-motor cortex related; DR, dorsal raphe nucleus; Gpe, Globus pallidus, external segment; LA, Lateral amygdalar nucleus; LDT, laterodorsal tegmental nucleus; LH, lateral hypothalamic area; LHb, lateral habenular nucleus; MEZ, hypothalamic medial zone; MHb. medial habenular nucleus; mPFC, posterior-medial frontal cortex; PO, posterior thalamic nuclear group; PVH, paraventricular hypothalamic nucleus; sAMY, Striatum-like amygdalar nuclei; VTA, ventral tegmental area; VP, ventral pallidum; ZI, zona incerta. The medial hypothalamus (MEZ) mainly includes the ventromedial nucleus of the hypothalamus (VMH) and the dorsomedial nucleus of the hypothalamus (DMH). Other brain regions definitions were as reported previously^23^. The brain regions (DORpm, DORsm, LH, PVH, MEZ, sAMY, LA, BLA, BMA) were outlined according to Allen Mouse Brain Atlas (https://mouse.brain-map.org/static/atlas). The other regions were outlined according to the Mouse Brain Atlas in Stereotaxic Coordinates, Franklin and Paxinos (2^nd^ ed).

### Quantification of Axonal Projection

For axonal fluorescent analysis, every fifth 40 μm coronal section was collected across the entire brain. 25 brain regions containing the major efferent targets of VTA were selected for analysis. The brain regions were outlined as described above. Quantification of axonal fluorescence intensities and the area of brain regions was performed by Fiji. For quantification of the axonal arborization area, the pixels three times higher than the gray-scale value of background were interpreted as signal from neuronal ensembles axons. Axonal arborization were calculated as the total area covered by the axonal fluorescence signal in a brain region. To quantify axonal fluorescence intensities, the integrated density of the axonal signal in a brain region as defined above was measured as the axonal fluorescence intensities for this region. The definition of Axonal Arborization per neuron: the axonal arborization area of each brain sites normalized by the number of EGFP^+^ cells in the VTA within each mouse. The definition of Axon Density: the ratio between axonal fluorescence intensities of each brain site and the intensities in the VTA within each mouse. The definition of Density Fraction: the fraction of axonal fluorescence intensities of each brain sites relative to total fluorescence intensities of all target sites within each mouse. Using Mor-Ens and Shock-Ens as class labels, targeted brain regions as variables, the supervised learning random forest algorithms were performed using the RandomForest in R to rank the importance of targeted brain regions distinguishing between Mor-Ens and Shock-Ens groups according to the mean Mean Decrease Gini coefficient.

### Real-Time Place Preference/Avoidance Test (RTPP/A)

All behavioral experiments were conducted during the light cycle of the day (8 a.m. to 8 p.m.). Mice were subjected to the RTPP/A test at 4 days after ensembles labeling. The RTPP/A apparatus was divided into two chambers (15 × 15 cm^2^). On day 1, mice were allowed to freely explore the two chambers for 20 min (pre-test). Mice that remained in one chamber for more than 65% of the total time were excluded from subsequent experiments. Day 2, the mice were returned to the apparatus. Optical stimulation (473 nm, 10 mW, 20 Hz, 5 ms) was delivered when the mice entered the paired chamber, and the light stimulation was stopped when the mice left the chamber. Mouse locations were tracked and analyzed using Ethovision XT software (Noldus, Wageningen, The Netherlands). Preference scores were defined as the time (in seconds) spent in the laser-paired chamber minus the time spent in the unpaired chamber. Bouts were defined as the frequency of entry into the laser-paired chamber.

### Conditioned Place Aversion (CPA) Test

Mice were subjected to the CPA test 4 days after ensemble labeling in the two-chamber apparatus with different tactile environments (Med-Associates, St. Albans, VT, USA). On day 1, mice were allowed to freely explore the two chambers for 20 min (pre-test). Mice that spent more than 65% of the total time in one chamber were excluded from subsequent experiments. Day 2-4, after 30 min of saline injection (4 mL/kg, i.p.), mice were confined to one chamber (saline-paired) for 30 min of conditioning. After 6 h, the mice were confined in another chamber (CNO-paired) for 30 min conditioning after 30 min injection of CNO (2 mg/kg, i.p.). On day 5, the mice were allowed to freely explore the two chambers for 20 min for testing. The CPA scores were defined as the time (in seconds) spent in the CNO-paired chamber minus the time spent in the saline-paired chamber.

### Elevated Plus Maze (EPM) Test

The elevated plus maze consisted of two open arms (length 34.5 cm. width 6.3 cm. height of the wall 0.8 cm) and two closed arms (length 34.5 cm. width 6.3 cm. height of the wall 19.5 cm). The four arms were perpendicular to each other. The height of the elevated plus maze was 75 cm. Mice were placed in the center (intersection of the four arms). The mice were allowed to freely explore the maze for 6 min. The maze was cleaned with 75% ethanol before each trail. The location of the mice was tracked and analyzed using Ethovision XT software (Noldus, Wageningen, The Netherlands). Bouts were defined as the frequency of entry into the open arms. For chemogenetics manipulation, the mice were injected with CNO (2 mg/kg, i.p.) 30 min before the test.

### Open field test

A laboratory-customized open field test box (length 42.0 cm, width 42.0 cm, height 40.0 cm) was used to assess the locomotor activity of mice. Mice were habituated in the room for at least 30 min before the test. During the test, each mouse was placed in the center of the open field and was allowed to explore freely for 30 min. Illumination was 50 lux. Video was recorded by a camera located above the box. The center zone was a central square area of 25% of the box. Time spent in the center zone and total distance were analyzed using Ethovision XT software.

### Ribosome Affinity Purification and RNA-Seq

The mixture of *AAV-RAM-d2TTA:: TRE-CRE and AAV-FLEX-NBL10* was injected into the VTA. Purification procedures after labeling ensembles were performed as previously reported^23^. Briefly, the brain tissues containing the VTA were rapidly dissected and immediately homogenized in ice-cold supplemented hybridization buffer (25 mM Tris [pH = 7.0], 25 mM Tris [pH = 8.0], 12 mM MgCl_2_, 100 mM KCl, 1% Triton X-100, 1 mM DTT, 100 μg/mL cycloheximide, 1 mg/mL heparin) containing protease inhibitors (04693159001, Roche, Switzerland) and RNase inhibitor (N2112S, Promega, USA). Each sample of VTA was enriched from two mice. Homogenates were centrifuged and the supernatant was incubated with 20 μL anti-HA antibody (H6908, Sigma-Aldrich) for 4 h at 4 ℃. 100 μL Dynabeads Protein G (#10003D, Novex, USA) was added to the supernatant and rotated overnight at 4 ℃. After washing with high-salt wash buffer (HSB), RNA was isolated from the Dynabeads by SuPerfectRI Total RNA Isolation Reagent according to the manufacturer's instructions. The library was prepared using the VAHTS mRNA-seq V3 Library Prep Kit (NR611, Vazyme Biotech Co., Ltd, Jiangsu, China) and sequenced on Illumina NovaSeq™ 6000 Sequencing System (Azenta, Jiangsu, China). RNA-seq data were submitted to NCBI Sequence Read Archive (SRA) under accession number PRJNA982298.

### Analysis of RNA-seq data

The raw data from Illumina were checked by FastQC and MultiQC 24. Trimmomatic 25 was used to remove the adapters and low-quality reads. STAR 26 software was used to map the reads to the mouse genome using the Ensembl GRCm39.106 gene annotation. FeatureCounts 27 was used for reads counting. DESeq2 28 was used for differential expression analysis. Genes with significant differences (adjusted p value < 0.05) were selected for further analysis. ClusterProfiler 29 was used for functional profiles analysis. STRING (version: 11.5, https://cn.string-db.org) was used to construct protein-protein interaction network.

### In-situ proximity ligation assay (PLA)

In-situ proximity ligation assay was performed using the Duolink Proximity Ligation Assay kit (DUO92008, Sigma-Aldrich). Primary antibodies: Anti-PLCβ3 Antibody (1:200, AP61288, Abcepta Biotech, USA), phosphor-AKT1^Thr308^ antibody (1:200, AP67522, Abcepta Biotech, USA). Briefly, 30 μm coronal brain sections were permeabilized with 0.3% Triton X-100 (in 1 × PBS buffer) for 30 min. The brain sections were mounted on the slides and then incubated with Duolink^®^ Blocking Solution (DUO92008, Sigma-Aldrich) in a heated humidity chamber for 60 min at 37 °C. Brain sections with primary antibodies diluted by Duolink^®^ Antibody Diluent (DUO92008, Sigma-Aldrich) at 4 °C overnight. After wash with buffers A (DUO82049, Sigma-Aldrich) for 2 × 5 min, the brain sections were incubated with anti-rabbit IgG PLUS PLA probes (DUO92002, Sigma-Aldrich) and MINUS PLA probes (DUO92005, Sigma-Aldrich) for 60 min at 37 °C. After wash with Buffers A, the brain sections were incubated with Duolink^®^ Ligation buffer (DUO92008, Sigma-Aldrich) for 30 min at 37 °C. After wash with buffers A, the brain sections were incubated with amplification solution (DUO92008, Sigma-Aldrich) for 100 min at 37 °C. After washing with buffers B (DUO82049, Sigma-Aldrich), the slides were mounted with coverslips with mounting medium (DUO82040, Sigma-Aldrich). Fluorescent images were captured by a Nikon-A1 confocal microscope (Tokyo, Japan) using 20 × air objective lens. The EGFP fluorescence of neurons was used to determine cell outlines. The pixels 1.5 times higher than the gray-scale value of background were interpreted as PLA signa, and PLA signal intensity (integrate density) 30 for each Mor-Ens cell or Shock-Ens cell were analyzed using ImageJ.

### Fiber photometry recording

200 μm-diameter 0.37 NA optic-fiber cannulas (Inper Tech, Hangzhou, Zhejiang, China) were implanted into the VTA of mice. The laser power at the tip of optical fiber was adjusted to 10 μW for 410 nm (isosbestic reference fluorescence) and 20 μW for 470 nm (Ca^2+^-dependent) by optical power meter (PM100D, Thorlabs, USA). Fluorescence signals were recorded by the Fiber Photometry System (QAXK-FPS-SS-LED, Thinker Tech Nanjing Biotech, Nanjing, Jiangsu, China). GCaMP signals were analyzed with MATLAB (R2019b, MathWorks) as previously reported 31. *ΔF/F* = (*F* - *F_0_*) */ F* and *F_0_* is the mean value of the pre-stimulus signal.

In the responses to the sucrose water, the mice deprived water for 12 h were allowed to freely explore the experiment chamber during fiber photometry recording. 2% sucrose water was delivered by the lick of the water spout. After 5 s of each licking, the water spout automatically retracts and can be licked again after an interval of 20 s. Each licking spout behavior triggers capacitive sensor (Thinker Tech Nanjing Biotech, Nanjing, Jiangsu, China) as TTL signal. The TTL signal and photometry signal were integrated simultaneously by the fiber photometry system (Thinker Tech Nanjing Biotech, Nanjing, Jiangsu, China). The video of mouse behaviors was recorded simultaneously. Photometry signals 2 s before each licking were recorded as the baseline. The first four times of licking behavior of each mouse were analyzed.

In the responses to tail suspension, tails of the mice were suspended for 3 min and the head of the mice was placed 20 cm from the bottom of home cage. The video was recorded simultaneously with fiber photometry recording. Photometry signals recorded 30 s before each suspension were the baseline. The mice were injected with Cariprazine (C126186, Aladdin, Shanghai, China) (0.2 mg/kg, i.p.) or UNC9994 (HY-117829, MedChemExpress, USA) (0.5 mg/kg, i.p.) 60 min prior to fiber photometry recording. Cariprazine and UNC9994 was dissolved in 0.9% saline.

### Quantification and Statistical Analysis

Sample sizes were based on previous studies 17, 31, 32. Data were analyzed using R and SPSS 22 (IBM, Armonk, NY, USA) and plotted using R. Comparisons of distribution data between two groups were analyzed using Kolmogorov-Smirnov tests. Comparisons between groups were analyzed by Student's* t* test (Unpaired, two-tailed), paired *t* test or Mann-Whitney U test. One-way ANOVA was used for the comparisons among multiple groups. Two-way repeated measures (RM) ANONA was used for the comparisons among multiple groups split on two variables followed by *Bonferroni's* post hoc test. Non-parametric tests were used when the assumptions of parametric tests were not met. Statistical significance was indicated as *P < 0.05, ** P < 0.01, *** P < 0.001. Data are presented as mean ± SEM.

## Results

### The spatial distribution of the VTA neuronal ensembles in response to morphine or to foot shock

**Figure 1:**
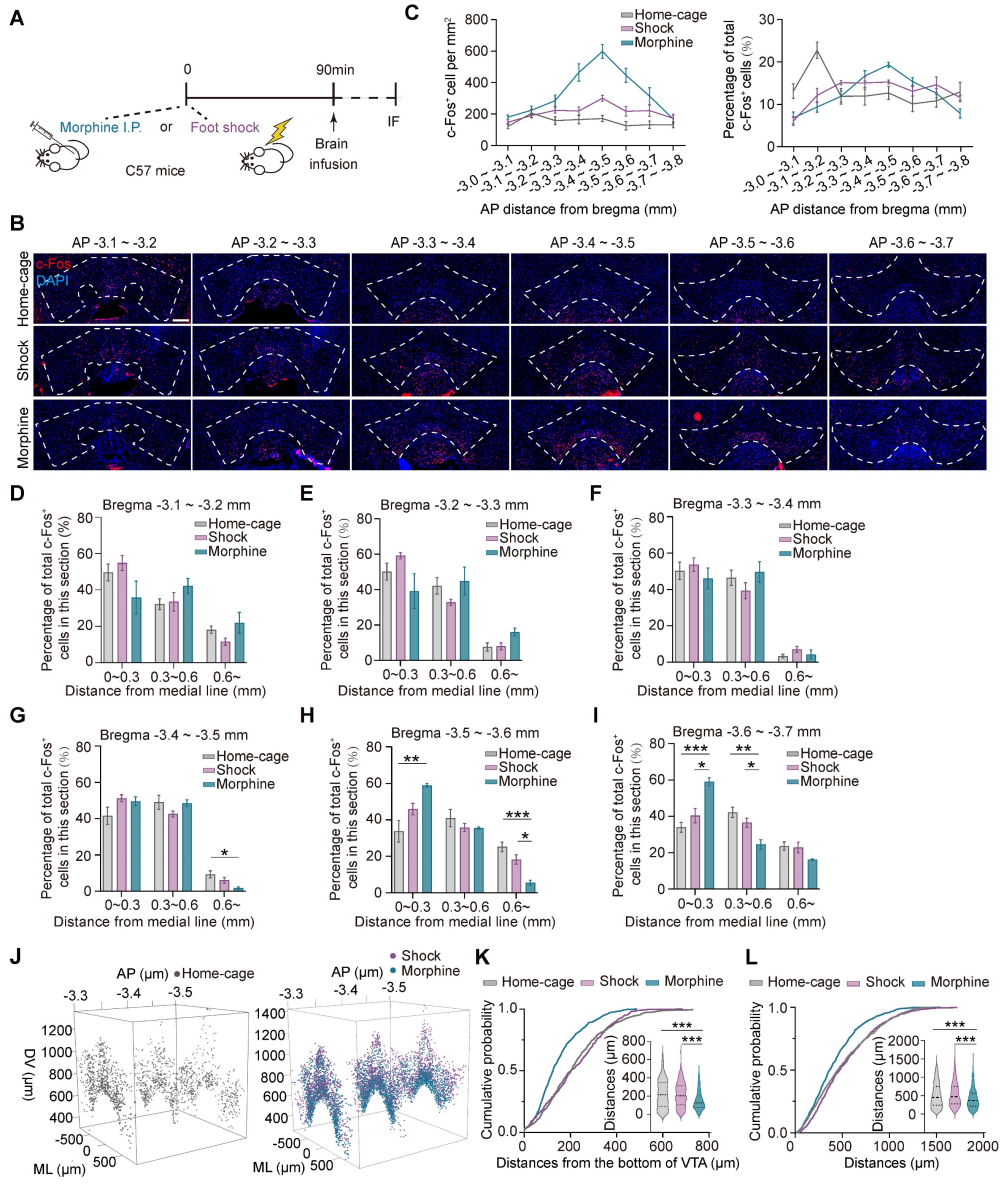
**The distinct distributions of the VTA ensembles in response to morphine or foot shock. (A)** Schematic of the experimental procedure for c-Fos staining. Mice were perfused and immunostained at 90 min after receiving morphine (10 mg/kg, i.p.), foot shocks (6 times, 0.75 mA, 2 s) or home-cage control. **(B)** Representative images of c-Fos immunostaining of the VTA along the anterior-posterior axis. Red: c-Fos; Blue: DAPI. Scale bar, 200 μm. Dashed lines: outline of the VTA. **(C)** Left, quantification of c-Fos^+^ cells (number per mm^2^) along the anterior-posterior axis in the VTA. Right, quantification of the percentage of c-Fos^+^ cells (normalized to the total number of c-Fos^+^ cells in the VTA). [Two-way RM ANOVA: Home-cage-Ens n = 7, Shock-Ens n = 6, Mor-Ens n = 4. Left: F _treatment × session_ (14, 92) = 11.28, P < 0.001; Right: F _treatment × session_ (14, 106) = 5.929, P < 0.001].** (D-I)** Quantification (% total counts in a section) of Mor-Ens, Shock-Ens and Home-cage-Ens along the mid-lateral axis of VTA. [Two-way RM ANOVA (D-I): (D) Home-cage-Ens n = 7, Shock-Ens n = 6, Mor-Ens n = 4, F _treatment × session_ (2.744, 19.206) = 5.929, P = 0.117. (E) F _treatment × session_ (2.545, 16.545) = 2.708, P = 0.086. (F) F _treatment × session_ (2.377, 15.448) = 2.708, P = 0.413. (G) F _treatment × session_ (4, 28) = 3.238, P = 0.026; Bonferroni post hoc, ML (0.6 ~) home-cage vs morphine P = 0.020. (H) Home-cage-Ens n = 5, Shock-Ens n = 6, Mor-Ens n = 4, F _treatment × session_ (4, 24) = 7.299, P = 0.001; Bonferroni post hoc, ML (0 ~ 0.3) home-cage vs morphine P = 0.004, ML (0.6 ~) home-cage vs morphine P = 0.001, ML (0.6 ~) morphine vs shock P = 0.011. (I) Home-cage-Ens n = 7, Shock-Ens n = 6, Mor-Ens n = 4, F _treatment × session_ (4, 28) = 9.277, P < 0.001; Bonferroni post hoc, ML (0 ~ 0.3) home-cage vs morphine P < 0.001, ML (0 ~ 0.3) shock vs morphine P = 0.007, ML (0.3 ~ 0.6) home-cage vs morphine P = 0.003, ML (0.3 ~ 0.6) shock vs morphine P = 0.043]. **(J)** Representative 3D distribution mapping of Home-cage-Ens, Mor-Ens and Shock-Ens in the VTA. Black dots: Home-cage-Ens; Purple dots: Shock-Ens; Blue dots: Mor-Ens. **(K)** Cumulative probability curves and the violin plots depict distributions of the distances from the bottom of the VTA (-3.5 mm). [Kruskal-Wallis H Test: Home-cage-Ens n = 270 cells from 7 mice, Shock-Ens n = 374 cells from 4 mice, Mor-Ens n = 542 cells from 4 mice, H = 77.457, P < 0.001; Bonferroni post hoc: home-cage vs morphine P < 0.001, shock vs morphine P < 0.001]. **(L)** Cumulative probability curves and the violin plot depict distributions of the distances between two c-Fos^+^ cells in the VTA (-3.5 mm) [Kruskal-Wallis H Test: Home-cage-Ens n = 1000 paired distances from 7 mice, Shock-Ens n = 1000 paired distances from 4 mice, Mor-Ens n = 1000 paired distances from 4 mice, H = 68.955, P < 0.001; Bonferroni post hoc: home-cage vs morphine P < 0.001, shock vs morphine P < 0.001]. *P < 0.05, **P < 0.01, ***P < 0.001. Data are shown as mean ± SEM.

The transcriptional levels of the immediate early genes (IEGs), such as* Arc, c-fos*, and* Creb, etc*. are rapidly and significantly increased during neuronal activation 33, 34, and therefore are often used to identify neuronal ensembles in response to specific experiences 22. To investigate the spatial distribution of the ensembles in response to pathological stimuli in the ventral tegmental area (VTA), C57BL/6 male mice were injected with morphine (10 mg per kg of body weight) or subjected to foot shocks (6 times, 0.75 mA, 2 s) in the same context. The control group consisted of mice in their home-cage without any manipulation. 90 min after the stimulation, the distributions of the neuronal ensembles in response to foot shock (shock neuronal ensembles, Shock-Ens), morphine (morphine neuronal ensembles, Mor-Ens) or home-cage (Home-cage-Ens) in the VTA were assessed by c-Fos staining (Figure 1A-B). The distribution of c-Fos^+^ cells in the home-cage group was relatively enriched in the rostral VTA (AP -3.1 ~ -3.2 mm) along the rostral-caudal axis of the VTA (Figure 1C right). However, the distribution of c-Fos^+^ cells in the morphine group was more concentrated in the middle of the VTA (AP -3.4 ~ -3.5 mm), while in the foot shock group, the distribution was more uniform along the rostral-caudal axis of the VTA (Figure 1C). Additionally, in the morphine group, the c-Fos^+^ cells were enriched near the midline in the caudal VTA (AP -3.5 ~ -3.7 mm) (Figure 1D-I).

To examine the distribution of c-Fos^+^ cells along the ventral-dorsal axis, the c-Fos^+^ cells mapped onto the three-dimensional coordinate system were analyzed in the middle part of the VTA (AP -3.3 ~ -3.6 mm) (Figure 1J). The cumulative probability distribution of the distance from the bottom of the VTA (AP -3.5 mm) showed that c-Fos^+^ cells in the morphine group were relatively enriched in the ventral part compared to the home-cage and foot shock groups (Figure 1K). The cumulative probability distributions of the distance between two paired c-Fos^+^ cells showed that c-Fos^+^ cells in response to morphine were relatively more concentrated than the neuronal ensembles in response to foot shock or home-cage (Figure 1L).

These results suggest that the spatial distribution of the VTA neuronal ensembles recruited by various stressors differed significantly. The distribution of Mor-Ens was more clustered and were relatively enriched in the ventral-caudal part of the VTA, whereas the distribution of Shock-Ens was dispersed throughout the VTA.

### Different behavior outcomes driven by Mor-Ens and Shock-Ens activation

**Figure 2:**
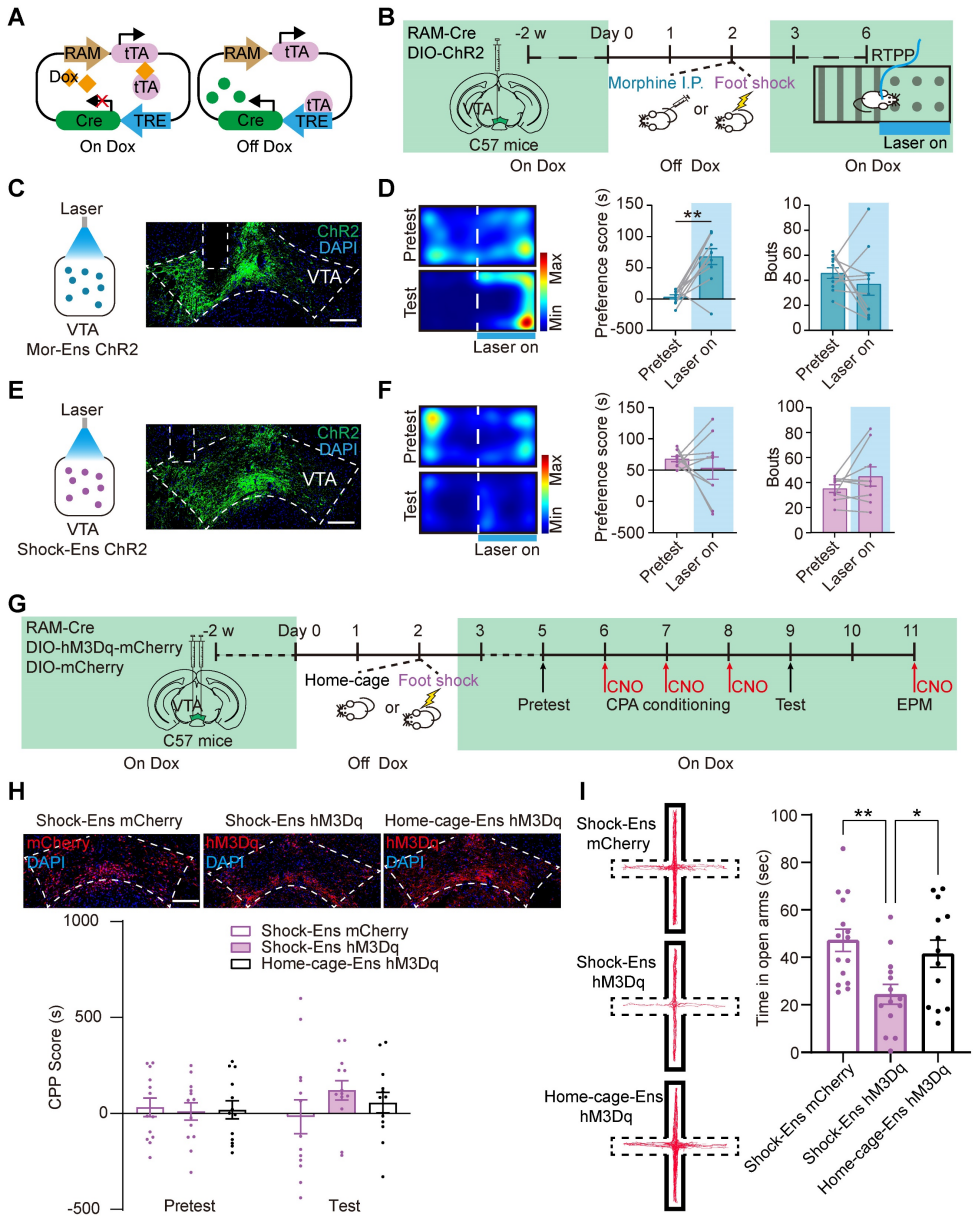
** Activation of the VTA Mor-Ens and VTA Shock-Ens lead to place preference and increased anxiety level, respectively. (A)** Diagram of the Cre-dependent Robust Activity Marking (CRAM) system.** (B)** Schematic of experimental procedure for ChR2 tagging in the VTA ensembles and the RTPP/A task. The mice received optogenetic stimulation (473 nm, 10 mW, 20 Hz, 5 ms) when entered the paired chamber. **(C, E)** Left, diagram of optogenetic activation of the VTA neuronal ensembles. Right, representative images of ChR2 or EGFP expressing in the VTA. Dashed lines: outline of the VTA and optic fiber trace. Green: EGFP; Blue: DAPI. Scale bar: 200 μm.** (D, F)** Left, representative heat maps of RTPP/A. Middle, quantification of preference score. Right, quantification of bouts (Frequency of entering the chamber paired with laser). [Paired t-test: (D) Middle, Mor-Ens n = 10, t (9) = -4.486, P = 0.002; Right, Mor-Ens n = 10, t (9) = 0.900, P = 0.392; (F) Middle, Shock-Ens n = 9, t (8) = 0.709, P = 0.498; Wilcoxon Signed Ranks Test: (F) Right, Shock-Ens n = 9, Z = -0.841, P = 0.400]. **(G)** Schematic of the experimental procedure for the virus injection and the behavior test. Mice were injected with CNO (2 mg/kg, i.p.) 30 min before the test. **(H)** Chemogenetic activation of the VTA Shock-Ens or Home-cage-Ens did not result in a preference/avoidance for the CNO-paired chamber. Top, representative images of hM3Dq or mCherry expressing in the VTA. Dashed lines: outline of the VTA. Red: hM3Dq or mCherry; Blue: DAPI. Scale bar: 200 μm. [Two-way RM ANOVA: Shock-Ens mCherry n = 13, Shock-Ens hM3Dq n = 13, Home-cage-Ens hM3Dq n = 13, F _treatment × session_ (2, 36) = 0.916, P = 0.409]. (I) Chemogenetic activation of Shock-Ens reduced the time spent in the open arms in the elevated plus maze (EPM) task. Left, Representative tracks during the 6 min EPM task. The areas surrounded by the solid line are closed arms. The areas surrounded by the dashed lines are open arms. The red line represents the mouse tracks. Right, time spent in the open arms. [One-way ANOVA: Shock-Ens mCherry n = 15, Shock-Ens hM3Dq n = 14, Home-cage-Ens hM3Dq n = 13, F _treatment × session_ (2, 39) = 6.022, P = 0.005; Bonferroni post hoc: Shock-Ens hM3Dq vs Shock-Ens mCherry, P = 0.004; Shock-Ens hM3Dq vs Home-cage-Ens hM3Dq, P = 0.039]. *P < 0.05, **P < 0.01, ***P < 0.001. Data are shown as mean ± SEM.

A single intraperitoneal (i.p.) injection of morphine induces a preference for the injection site, suggesting a reward effect of morphine 16, whereas foot shock induces avoidance behavior, anxiety and freezing in mice 13, 16. To investigate the place preference/avoidance behaviors driven by Mor-Ens and Shock-Ens, a Cre-dependent robust activity-regulated promoter (CRAM) 22 was used to label and manipulate neuronal ensembles in response to specific experiences (Figure 2A). A previous study has confirmed the sensitivity and specificity of the CRAM system 17. In this study, mice were infected with *AAV-RAM-tTA-TRE-Cre* and *AAV-DIO-ChR2-EYFP* in the VTA, and implanted with an optical fiber over the VTA. ChR2 proteins were expressed in Mor-Ens or Shock-Ens before the preference/avoidance task (RTPP/A) (Figure 2B). The mice were then received optical stimulation (473 nm, 10 mW, 5 ms, 20 Hz) on one side and no stimulation on the other side. Optogenetic activation of the VTA Mor-Ens resulted in a preference for the laser-paired side (Figure 2C-D). However, optogenetic activation of VTA Shock-Ens did not induce any significant avoidance behavior (Figure 2E-F). The frequency of entering the laser-paired chamber between the pretest and test sessions was not significantly different in both Mor-Ens and Shock-Ens groups (Figure 2D right, 2F right). These results suggest that activation of VTA Mor-Ens produces a place preference effect.

A previous study showed that chemogenetic activation of VTA Mor-Ens resulted in a preference for the CNO-paired chamber and increased time spent in the open arms during the elevated plus maze test, while chemogenetic activation of the VTA ensembles in response to saline (Sal-Ens) did not induce such effects 17. To investigate the effect of chemogenetic activation of Shock-Ens, the C57 mice were infected with *AAV-RAM-tTA-TRE-Cre, AAV-DIO-hM3Dq-mCherry or AAV-DIO-mCherry* in the VTA to express hM3Dq or mCherry in Shock-Ens or Home-cage-Ens (randomly labeled ensembles) (Figure 2G). C-Fos staining revealed that CNO significantly activated hM3Dq^+^ cells in the VTA (Figure S1B). The number of RAM-tagged hM3Dq^+^ Shock-Ens or Home-cage-Ens was not significantly different (Figure S1C). The result of the conditioned place preference (CPP) test showed that chemogenetic activation of Shock-Ens or Home-cage-Ens did not lead to a preference for the CNO-paired chamber (Figure 2H). In the elevated plus maze test, chemogenetic activation of Shock-Ens resulted in a decrease in the time spent in the open arms compared to the home-cage group (Figure 2I). These results suggest that the activation of the VTA Shock-Ens increases the anxiety level of mice.

**Figure 3:**
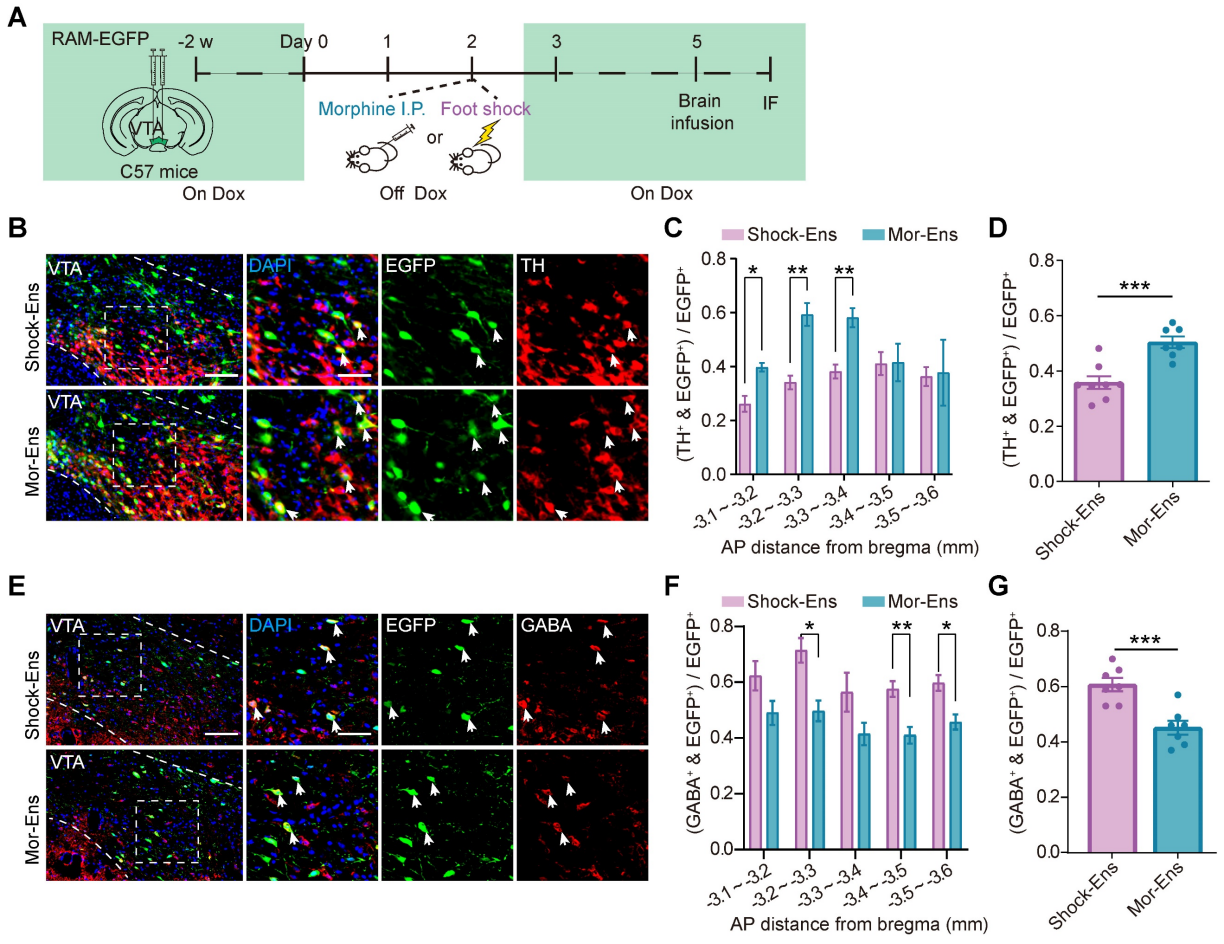
** Mor-Ens and Shock-Ens in the VTA contain different proportions of dopaminergic and GABAergic neurons. (A)** Schematic of the experimental procedure for immunofluorescence staining. IF: immunofluorescence** (B)** Representative images of TH staining in the VTA. Green: EGFP; Red: TH. Scale bar: left 100 μm, right 50 μm. The dashed line represents the boundary of the VTA and the enlarged area. White arrows indicate the TH^+^ EGFP^+^ cells.** (C)** The percentage of TH^+^ cells in EGFP^+^ ensembles along rostral-caudal axis of the VTA. [Two-way RM ANOVA: Shock-Ens n = 8, Mor-Ens n = 7, F _treatment × session_ (4, 39) = 3.857, P < 0.001. Bonferroni post hoc: ML (-3.1, -3.2), P = 0.013; ML (-3.2, -3.3), P = 0.002; ML (-3.3, -3.4), P = 0.004].** (D)** The percentage of TH^+^ cells in EGFP^+^ ensembles in the VTA. [Two-tailed Student's t-test: Shock-Ens n = 8, Mor-Ens n = 7, t (13) = -4.645, P < 0.001]. **(E)** Representative images of GABA staining in the VTA. Green: EGFP; Red: GABA. Scale bar: left 100 μm, right 50 μm. The dashed line represents the boundary of the VTA and the enlarged VTA area. White arrows indicate the GABA^+^EGFP^+^ cells. **(F)** The percentage of GABA^+^ cells in EGFP^+^ ensembles along rostral-caudal axis of the VTA. [Two-way RM ANOVA: (F) Shock-Ens n = 7, Mor-Ens n = 7, F _treatment × session_ (4, 48) = 0.438, P = 0.780, F _ensembles_ (1, 12) = 17.848, P = 0.001. Bonferroni post hoc: ML (-3.2, -3.3), P = 0.014; ML (-3.4, -3.5), P = 0.008; ML (-3.5, -3.6), P = 0.019]. **(G)** The percentage of GABA^+^ cells in EGFP^+^ ensembles in the VTA. [Two-tailed Student's t-test: Shock-Ens n = 7, Mor-Ens n = 7, t (12) = 4.433, P = 0.001]. *P < 0.05, **P < 0.01, ***P < 0.001. Data are shown as mean ± SEM.

Dopaminergic and GABAergic composition between Mor-Ens and Shock-Ens were further investigated. Mice were infected with *AAV-RAM-tTA-TRE-EGFP* in the VTA. Shock-Ens and Mor-Ens were labeled with EGFP (Figure 3A). Tyrosine hydroxylase (TH, a marker for dopaminergic neurons) and gamma-aminobutyric acid (GABA) staining was performed. The percentage of TH^+^ population in EGFP^+^ cells was higher in the Mor-Ens than in the Shock-Ens in the VTA (Mor-Ens vs Shock-Ens, 50.4 ± 2.1% vs 35.6 ± 2.3%) (Figure 3C-D), whereas the percentage of GABAergic neurons was lower in the Mor-Ens than in the Shock-Ens (Mor-Ens vs Shock-Ens, 45.1 ± 2.5% vs 60.7 ± 2.4%) (Figure 3F-G). Taken together, these results suggest that the VTA Mor-Ens and Shock-Ens have different dopaminergic or GABAergic compositions. Activation of the VTA Mor-Ens may drive approach behavior, while activation of the Shock-Ens may lead to anxiety-like behaviors.

### Brain-wide mapping of monosynaptic afferents to the VTA Mor- and Shock-Ens

**Figure 4:**
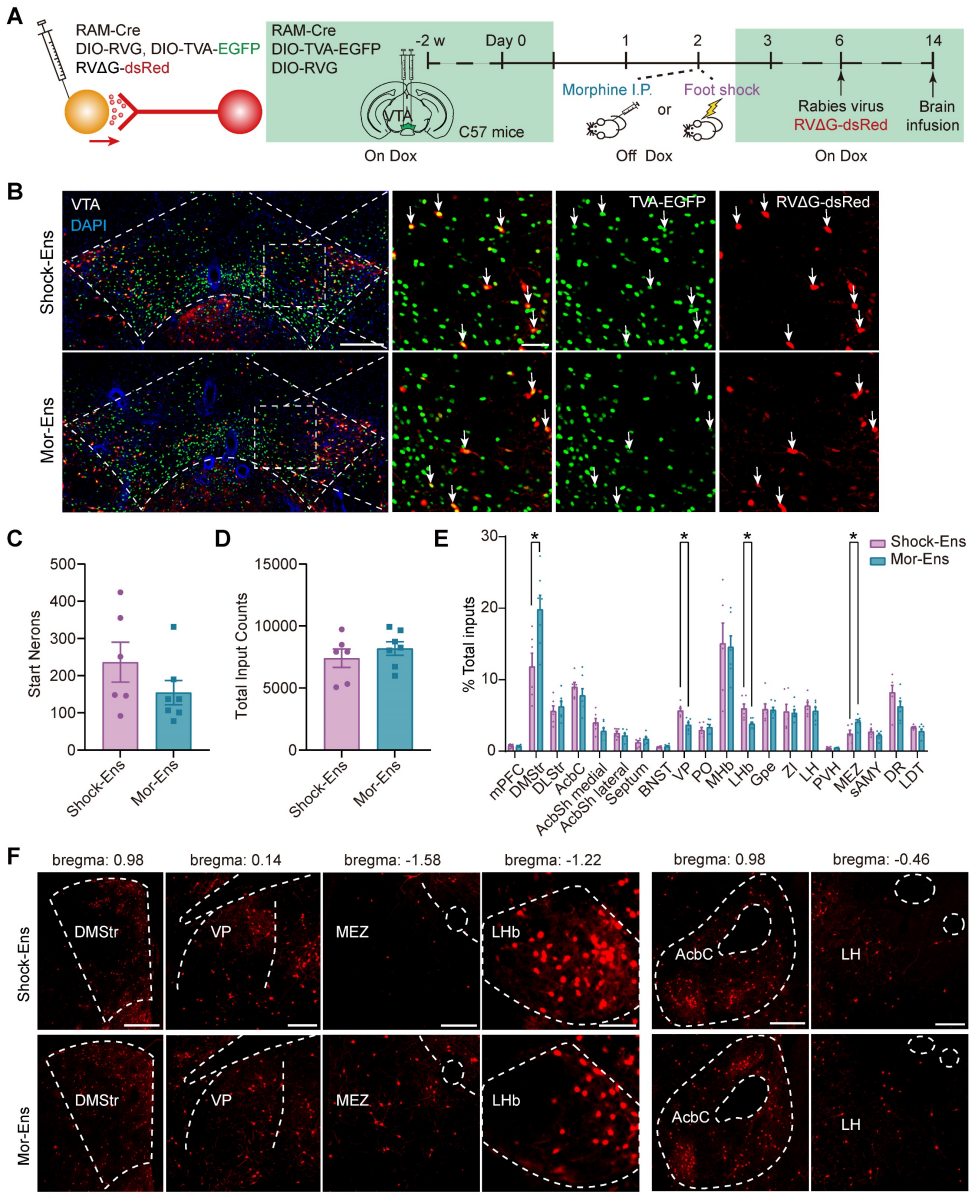
** Rabies virus-mediated monosynaptic tracing of the inputs onto Mor-Ens or Shock-Ens in the VTA. (A)** Schematic of the rabies-mediated monosynaptic retrograde labeling of the inputs onto VTA Mor-Ens or Shock-Ens. Mice were injected with dsRed-expressing rabies virus after Mor-Ens or Shock-Ens labeling and the brains were collected 7 days later.** (B)** Representative confocal images of the VTA starter neurons. Green: TVA-EGFP; Red: RVΔG-dsRed; Blue: DAPI. Scale bars: left 200 μm, right 50 μm. Dashed lines: outline of VTA. White arrows indicate EGFP^+^dsRed^+^ starter neurons in the VTA. **(C)** Quantification of the total number of starter neurons (EGFP^+^dsRed^+^) in the VTA. [Two-tailed Student's t-test: Shock-Ens n = 6, Mor-Ens n = 7, t (11) = 1.347, P = 0.205]. **(D)** Quantification of the inputs (dsRed^+^ cells) in 20 brain regions. [Two-tailed Student's t-test: Shock-Ens n = 6, Mor-Ens n = 7, t (11) = 0.861, P = 0.408]. **(E)** DsRed^+^ inputs of each brain region relative to total dsRed^+^. [Two-way RM ANOVA: Shock-Ens n = 6, Mor-Ens n = 7, F _treatment × session_ (4.417, 48.592) = 2.927, P = 0.026, Fisher's LSD post hoc: DMStr, P = 0.014; VP, P = 0.002; LHb, P = 0.015; MEZ, P = 0.020]. **(F)** Representative images of the inputs (dsRed^+^ cells) from selected brain regions. Red: dsRed. Dashed lines: outline of brain regions. Scale bars, 500 μm for DMStr; 200 μm for VP and MEZ; 100 μm for LHb; 250 μm for Acbc and LH. *P < 0.05, **P < 0.01, ***P < 0.001. Data are shown as mean ± SEM.

The rabies virus-based and ensembles-specific monosynaptic tracing techniques allow us to label monosynaptic inputs with ensemble specificity 9. To compare the whole brain map of the inputs to VTA Mor-Ens and Shock-Ens, mice were infected with *AAV-RAM-tTA-TRE-Cre* and Cre-dependent helper viruses *AAV-DIO-TVA-EGFP, AAV-DIO-RVG* in the VTA. One week after labeling with Mor-Ens or Shock-Ens, mice were infected with the modified rabies virus *RV-EvnA-△G-dsRed* in the VTA. After one week, the mice were sacrificed, and fluorescence imaging was performed to analyze the distribution of dsRed-positive neurons (input neurons) in different brain regions (Figure 4A). The starter ensembles expressing EGFP and dsRed were restricted to the VTA (Figure 4B), and the total numbers of the starter neurons (EGFP^+^ dsRed^+^ cells in the VTA) and input neurons (dsRed^+^ cells) were not significantly different between the Mor-Ens and Shock-Ens groups (Figure 4C-D). The normalized input index, which represents the ratio of dsRed^+^ cells to all input neurons, showed that Mor-Ens received more inputs from the dorsal medial striatum (DMStr) and hypothalamic medial zone (MEZ), and fewer inputs from the ventral pallidum (VP) and lateral habenular nucleus (LHb) compared to the Shock ensemble (Figure 4E-F). DMStr, MEZ, VP and LHb have been implicated in emotional and motivational processing 35-38. The data suggests that Mor-Ens and Shock-Ens in the VTA establish different connections with the brain regions involved in diverse emotional processing.

### The zona incerta (ZI) and ventral pallidum (VP) received more afferents from Mor-Ens than from Shock-Ens

**Figure 5:**
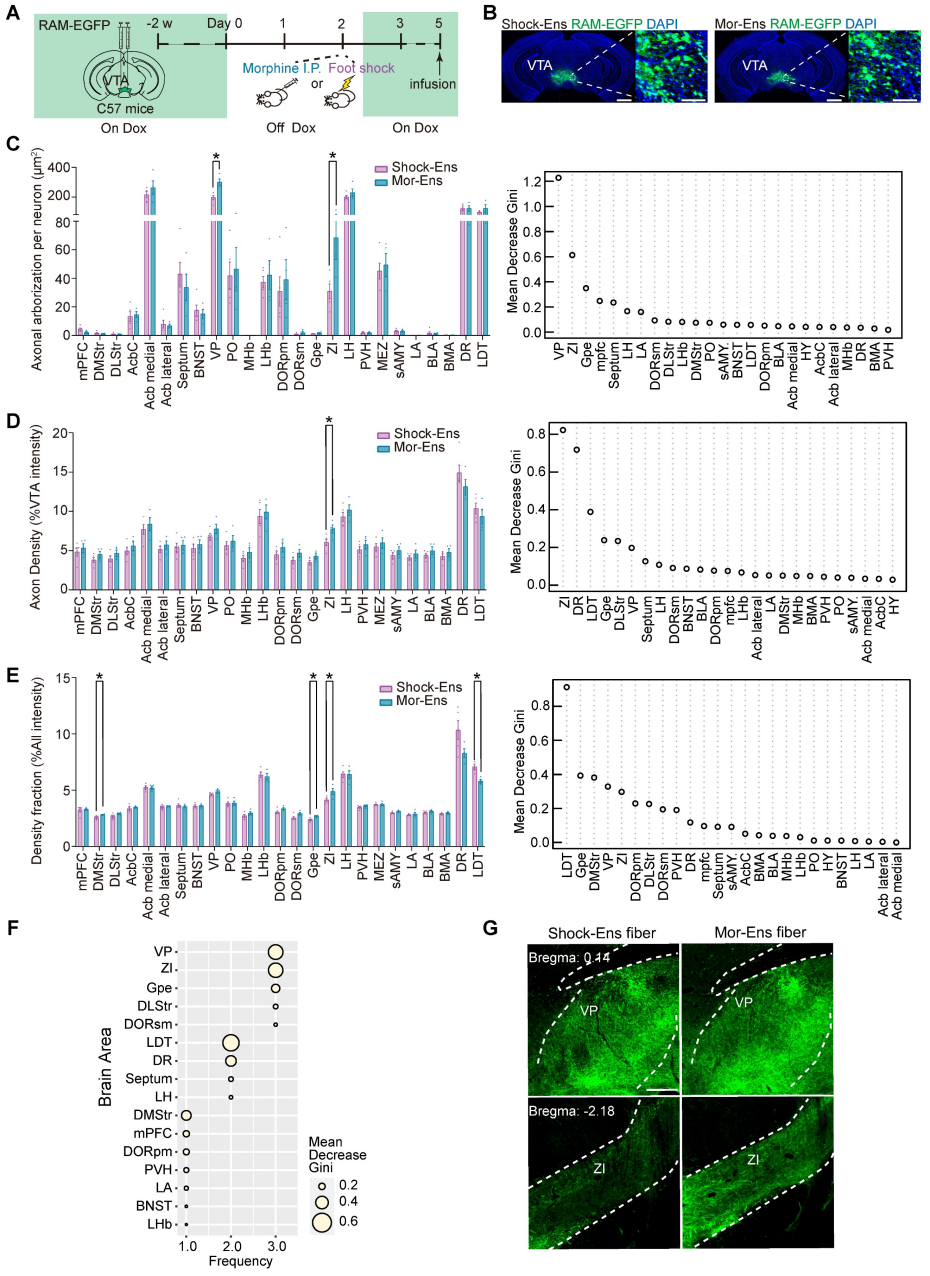
** ZI and VP receive more afferences from Mor-Ens than from Shock-Ens. (A)** Schematic of the experimental procedure to label the Mor-Ens or Shock-Ens in the VTA. **(B)** Representative images of EGFP^+^ ensembles in the VTA. Green: EGFP; Blue: DAPI. Scale bar: left 1000 μm, right 100 μm. **(C-E)** Left: quantitative analysis of the axonal projections from VTA Mor-Ens and Shock Ens in 25 target brain regions. Right: the importance ranking of 25 target brain regions based on random forest algorithm. [Two-tailed Student's t-test: Shock-Ens n = 5, Mor-Ens n = 4, (C) VP: t (7) = -4.364, P = 0.003, ZI: t (7) = -2.563, P = 0.037; (D) ZI: t (7) = -2.599, P = 0.035; (E) Gpe: t (7) = -3.406, P = 0.011, ZI: t (7) = -2.673, P = 0.032, LDT: t (7) = 4.242, P = 0.004]. **(F)** Integrated evaluation of the importance for the targeting brain regions. Frequency: The number of times the brain regions ranked in the top 10 on each index. Size of dots: the average Mean Decrease Gini. **(G)** Representative images of axonal projections in VP and ZI. Green: EGFP. Scale bar: 250 μm. Dashed lines: outline of brain regions. *P < 0.05, **P < 0.01, ***P < 0.001. Data are shown as mean ± SEM.

Previously studies have suggested that VTA neurons projecting to different brain regions possess biased inputs, and VTA dopaminergic neurons with different functions display different axonal projections 9, 39. To investigate the axonal projections of Mor-Ens and Shock-Ens, mice were infected with *AAV-RAM-tTA-TRE-EGFP* in the VTA to express EGFP in Mor-Ens or Shock-Ens (Figure 5A-B). Axonal fluorescence intensities and axonal arborization area were analyzed in 25 brain regions, which include the major targets of the VTA projections. Three indexes were used to describe the output characteristics. Axonal arborization per neuron (the axonal arborization area of each brain region normalized to the number of EGFP^+^ cells in the VTA) (Figure 5C left), axon density (the ratio of axonal fluorescence intensities of each brain site and the VTA fluorescence intensities) (Figure 5D left), and density fraction (the fraction of axonal fluorescence intensities of each brain site relative to the total fluorescence intensities of all target sites) (Figure 5E left). Supervised learning random forest algorithms were used to rank the importance of the targeted brain regions for each of the three indexes. The algorithms distinguished between the Mor-Ens and Shock-Ens groups based on the Mean Decrease Gini coefficient. A larger Mean Decrease Gini indicates a more significant role of the brain region in discriminating between the two groups (Figure 5C-E right). To comprehensively utilize these three indexes, the top 10 brain regions for each index were selected and their frequency of occurrence and average Mean Decrease Gini were calculated (Figure 5F). Among these regions, ZI and VP had the highest frequency (3) and Mean Decrease Gini values (ZI: 0.58, VP: 0.59) (Figure 5F). These results suggest that ZI and VP turn out to be the two target sites with the largest differences in the outputs of Mor-Ens and Shock-Ens. Taken together, these results suggest that neuronal ensembles in the VTA recruited by different stressors may exhibit anatomical heterogeneity, with different input and output connectivity.

### Differential expression of the genes encoded receptors and GPCR signaling pathways in VTA Mor-Ens and Shock-Ens

**Figure 6:**
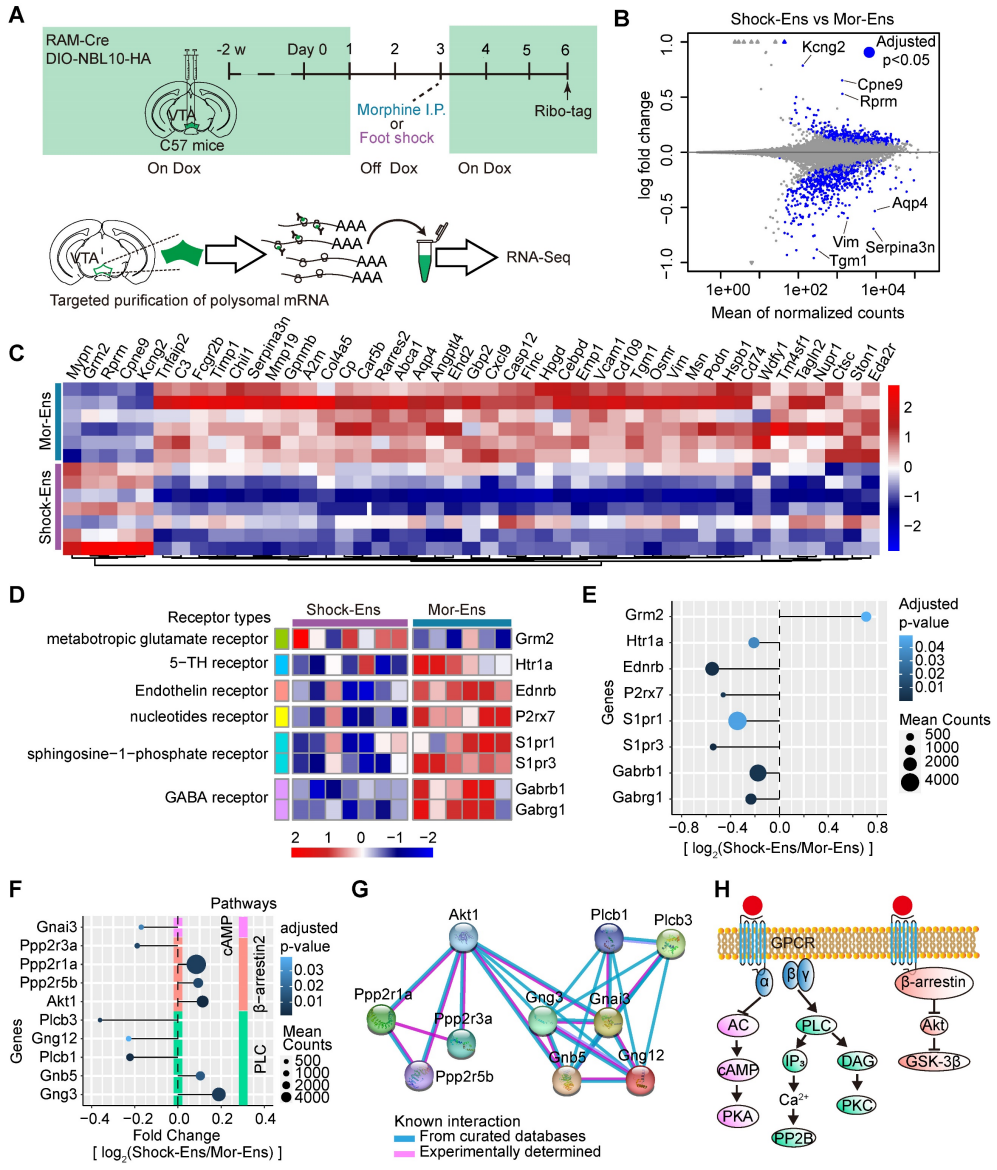
** Different molecular features of the VTA Mor-Ens and Shock-Ens. (A)** Experimental scheme of purifying ribosome-associated mRNAs and sequencing of the VTA Mor-Ens or Shock-Ens. **(B)** An MA-plot with the log_2_-fold changes (Shock-Ens vs Mor-Ens) over the mean of normalized counts. Points with adjusted p-value < 0.05 were colored blue. **(C)** Heatmap of differentially expressed genes (DEGs: fold change > 1.5 and adjusted p value < 0.05) (Shock-Ens n = 7 sample from 14 mice. Mor-Ens n = 6 sample from 12 mice). **(D)** Heatmap of differentially expressed genes encoding neuroactive receptors (adjusted p-value < 0.05). **(E)** Dot plot for DEGs of neuroactive receptors. The size of the dots represents the gene mean counts and the color of the dots represents the adjusted p-value.** (F)** Dot plot for DEGs involved in GPCRs signaling pathways. The size of the dots represents the gene mean counts; the color of the dots represents the adjusted p-value. **(G)** The protein-protein interaction networks for the DEGs involved in GPCR signaling pathways were generated from the STRING database (version: 11.5, https://cn.string-db.org). **(H)** An example of the G protein-coupled receptors (GPCRs) G-protein signaling pathways and GPCRs/β-arrestin/Akt/glycogen synthase kinase 3 (GSK-3) signaling pathway.

The molecular profiling of the VTA Mor-Ens or Shock-Ens was further evaluated. The viral mixture of* AAV-RAM-tTA-TRE-Cre and AAV-Ef1α-DIO-NBL10-HA* was delivered into the VTA of mice. After tagging the NBL10 in the Mor-Ens and Shock-Ens, ribosome-associated transcripts in the ensembles were isolated for sequencing (Figure 6A). Analysis of the ribosome-associated transcripts in these two groups identified 1091 differentially expressed genes (DEGs) with adjusted p < 0.05. The Mor-Ens group (n = 6) had 613 genes that were relatively highly expressed, while the Shock-Ens group (n = 7) had 478 genes that were relatively highly expressed (Figure 6B). Among the DEGs, 45 genes showed more than 1.5-fold change (Shock-Ens vs Mor-Ens), with 5 genes being more abundant in Shock-Ens and 40 genes being more abundant in Mor-Ens (Figure 6C). VTA neurons receive afferents with various neurotransmitters that convey different emotional signals 7. Therefore, the sequencing data were further analyzed for differentially expressed transcripts of neuroactive receptors (Figure 6D). The transcript level of *Grm2*, the gene encoding the glutamate metabotropic receptor 2, was higher in Shock-Ens (Figure 6E). Transcripts for *Htr1a, Ednrb, P2rx7, S1pr1, S1pr3, Gabrb1, Gabrg1* were more abundant in Mor-Ens (Figure 6E). The proteins encoded by *Grm2*, *Htr1a, Ednrb, S1pr1,* and *S1pr3,* are belong to the family of G protein-coupled receptors (GPCRs). GPCRs convert extracellular stimuli into intracellular signals through G protein-dependent signaling pathways (activating G protein to regulate cyclic AMP, phospholipids or ion channels) and G-protein-independent pathways (interacting with arrestins). In addition to desensitizing GPCR signaling, there is accumulating evidence that β-arrestins initiate independent signaling pathways.

β-arrestin1 or β-arrestin-2 can form a complex with tyrosine-protein kinase Src (c-Src), signal-regulating kinase 1 (ASK1), mitogen-activated protein kinase MAPK kinase 4 (MKK4) and c-Jun N-terminal kinases (JNK), resulting in the activation of JNK3, ERK and promoting cell proliferation, *etc.* 40. Activated D2 receptors recruit β-arrestin2 to inhibit the activation of protein kinase B (AKT) by dephosphorylating their regulatory threonine residue (threonine 308), leading to the activation of glycogen synthase kinase 3 (GSK-3) 41, 42. The transcript level of genes involved in these GPCR signaling pathways were further analyzed. Ten genes were identified as differentially expressed in GPCR signaling pathways: *Gnai3, Ppp2r3a, Ppp2r1a, Ppp2r5b, Akt1, Plcb3, Gng12, Plcb1, Gnb5,* and* Gng3* involved in GPCR signaling pathways were identified (adjusted p < 0.05) (Figure 6F). The protein-protein interaction networks of these genes suggested that *Akt1, Plcb3,* and *Plcb1* may be the hub genes in this network (Figure 6G-H).

**Figure 7:**
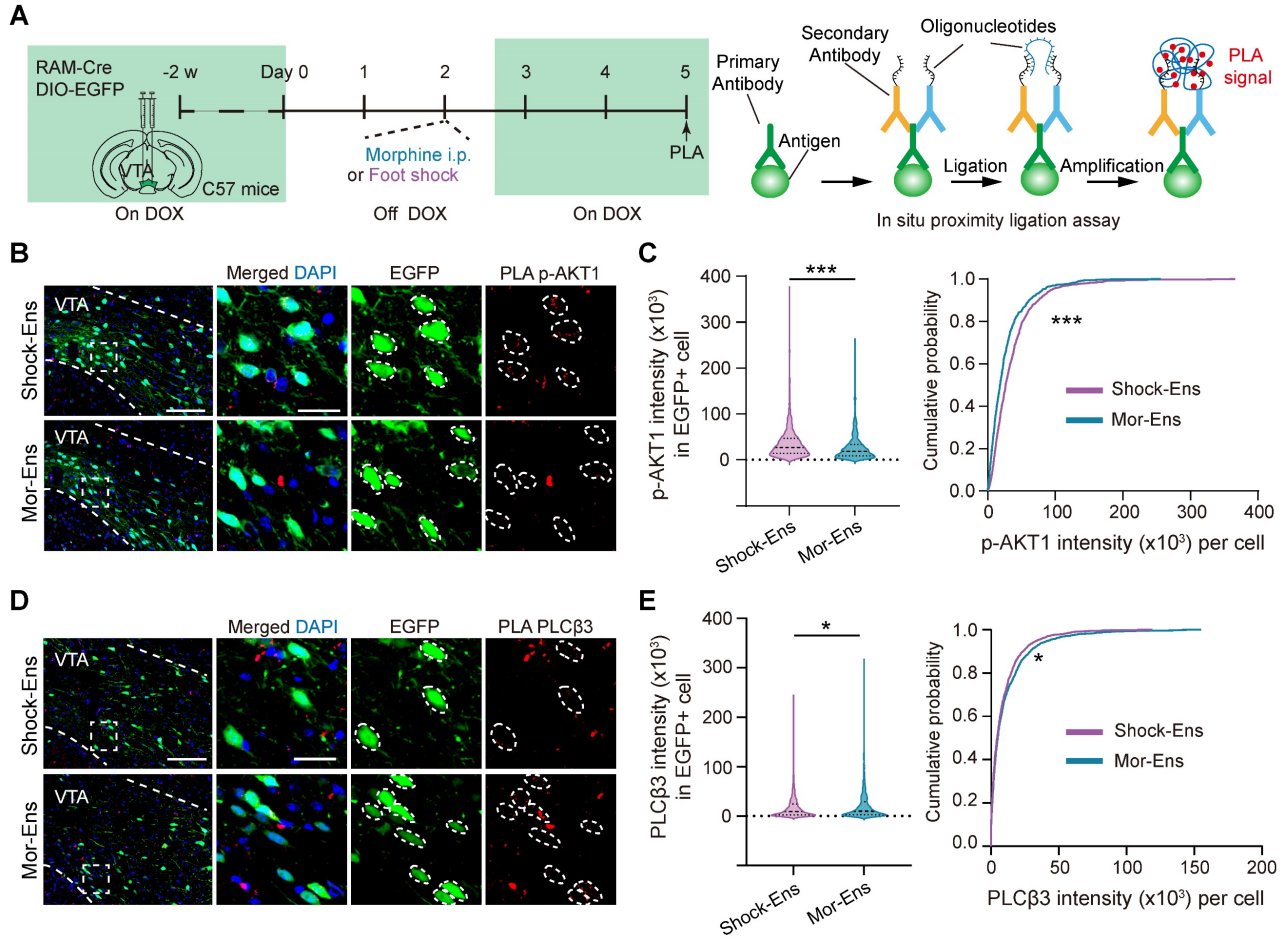
** The phosphorylated-AKT1^Thr308^ level is relatively higher in Shock-Ens, while the expression of PLCβ3 is relatively higher in Mor-Ens. (A)** Left, schematic of the experimental procedure for the in-situ proximity ligation assay. Right, the working principal diagram of PLA. **(B, D)** Representative images of PLA signaling for phosphor-AKT1^Thr308^ or PLCβ3 in the VTA. Green: EGFP; Red: p-AKT1 or PLCβ3. Scale bar: left 100 μm, right 25 μm. Dashed lines: outline of EGFP^+^ cell. **(C, E)** The violin plot and cumulative probability curves illustrate the distribution of the PLA signal intensity in each EGFP^+^ cell. [Mann-Whitney test: (C) Left, Shock-Ens n = 822 cells from 5 mice, Mor-Ens n = 754 cells from 5 mice, U = 240371, P < 0.001; (E) left, Shock-Ens n = 1340 cells from 8 mice, Mor-Ens n = 1415 cells from 8 mice, U = 898116, P = 0.017. Kolmogorov-Smirnov test: (C) right, D = 0.179, P < 0.001; (E) right, D = 0.056, P = 0.026]. *P < 0.05, **P < 0.01, ***P < 0.001. Data are shown as mean ± SEM.

The in-situ proximity ligation assay (PLA) was used to enhance the efficiency of in situ protein analysis of PLCβ3 and phosphor-AKT1^Thr308^ in the VTA (Figure 7A). The fluorescent signal intensity of phosphor-AKT1^Thr308^ were relatively higher in the VTA Shock-Ens than in the VTA Mor-Ens (Figure 7B, 7C). Instead, the signal intensity of PLCβ3 in the VTA Shock-Ens was relatively lower than in the VTA Mor-Ens (Figure 7D-E). The PLA results were consistent with transcriptome sequencing. The result indicates that VTA Mor-Ens and Shock-Ens may possess different molecular signatures that are relatively enriched in G-protein- and β-arrestin-biased GPCR signaling pathways (Figure 6H).

### G-protein-biased agonist cariprazine increased the response of Mor-Ens to sucrose water and decreased the anxiety level during morphine withdrawal, whereas the β-arrestin-biased agonist UNC9994 alleviated the suppression of Shock-Ens by tail suspension

**Figure 8:**
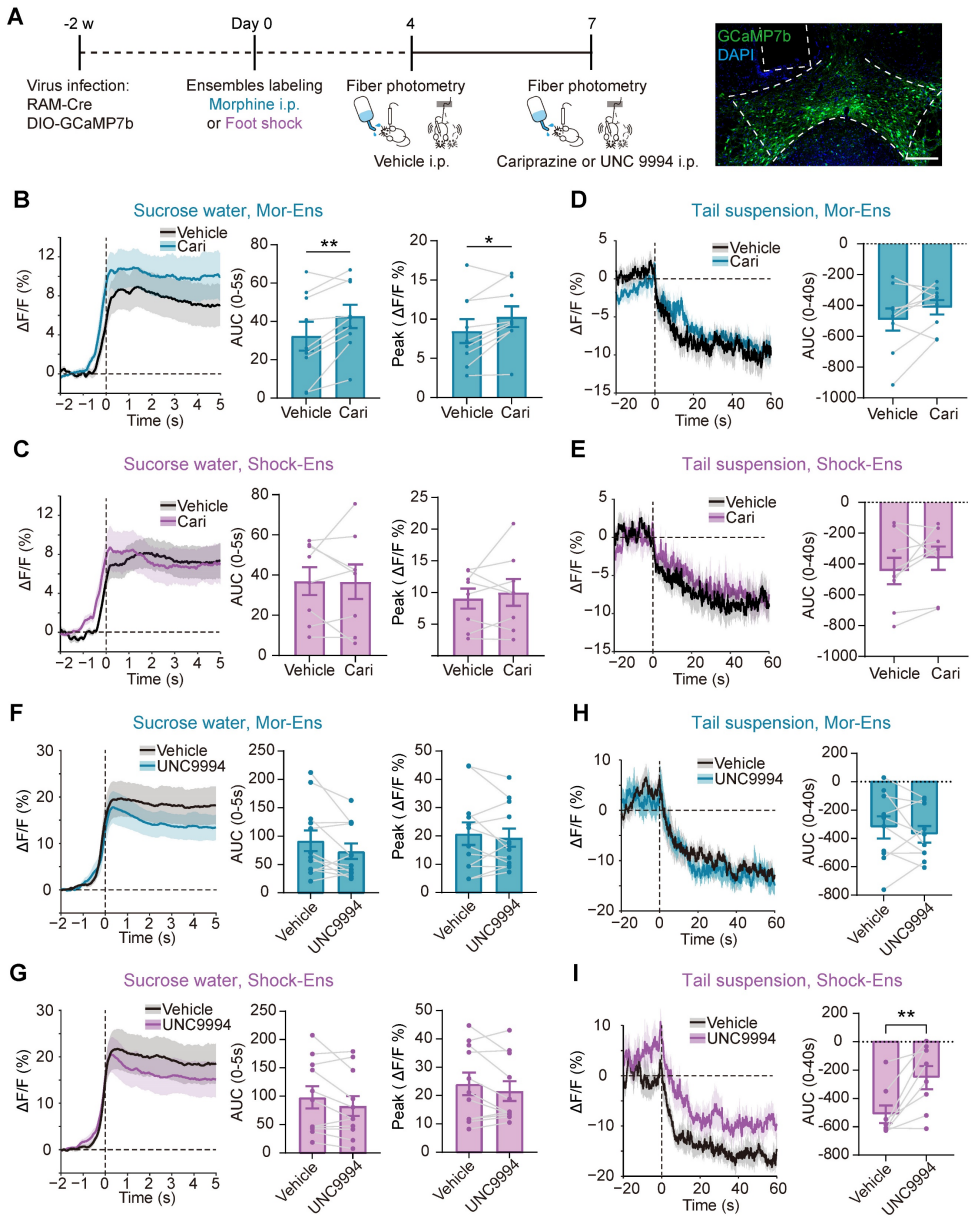
** Cariprazine increases the response of the Mor-Ens to sucrose water, while UNC9994 alleviates the response of Shock-Ens to tail suspension. (A)** Schematic of fiber photometry recording for VTA ensembles response to 2% (w/v) sucrose water or to tail suspension. The mice were intraperitoneally injected with cariprazine (0.2 mg/kg) or the UNC9994 (0.5 mg/kg) 60 min before the recording. Right, representative images of GCaMP7b expressing in the VTA. Dashed lines: outline of the VTA and optic fiber trace. Green: GCaMP7b; Blue: DAPI. Scale bar: 200 μm. **(B, C, F, G)** Left, the fiber photometry signal of the VTA ensemble responses to sucrose water. Solid lines represent the mean value. Gray shadows represent SEM. Middle, Area under the calcium photometry curve during sucrose water consumption (0-5 s). [Paired t-test: (B) n = 9, t (8) = -3.494, P = 0.008; (C) n = 8, t (7) = 0.061, P = 0.953; (F) n = 12, t (11) = 1.876, P = 0.087; (G) n = 11, t (10) = 1.984, P = 0.075]. Right, Average peak of photometric signal responses to sucrose water. [Paired t-test: (B) n = 9, t (8) = -2.845, P = 0.022; (C) n = 8, t (7) = -0.712, P = 0.500; (F) n = 12, t (11) = 0.757, P = 0.465; (G) n = 11, t (10) = 1.507, P = 0.163]. **(D, E, H, I)** Left, the fiber photometry signal of VTA ensembles responses to tail suspension. Solid lines represent the mean value. Gray shadows represent SEM. Right, Area under calcium photometry curve during tail suspension (0-40 s). [Paired t-test: (D) n = 9, t (8) = -1.565, P = 0.156; (E) n = 8, t (7) = -1.396, P = 0.205; (H) n = 10, t (9) = 0.852, P = 0.416; (I) n = 8, t (7) = -3.714, P = 0.008]. *P < 0.05, **P < 0.01, ***P < 0.001. Data are shown as mean ± SEM.

To test whether G protein and β-arrestin signaling pathways were differentially affect in the response of Mor-Ens and Shock-Ens to reward or stress, mice were infected with *AAV-RAM-tTA-TRE-Cre and AAV-DIO-GCaMP7b* in the VTA (Figure 8A). The calcium dynamics of Mor-Ens or Shock-Ens in mice was recorded by the fiber photometry. In response to sucrose water, both Mor-Ens and Shock-Ens showed increased neuronal activity (Figure 8B-C), while their activity decreased in response to tail suspension (Figure 8D-E). The 410 nm signal channel showed no significant change, indicating that the calcium signals were not caused by movement-induced interface loosening (Figure S2). Intraperitoneal injection of cariprazine (0.2 mg/kg), an D2/D3 receptor partial agonist with a more potent agonist effect on the G protein signaling pathway and an antagonist effect on the dopamine receptor β-arrestin2 signaling pathway 43, 44, increased the activity of VTA Mor-Ens but not the Shock-Ens in response to sucrose water (Figure 8B-C), but did not affect the responses of VTA Mor-Ens and Shock-Ens to tail suspension (Figure 8D-E). Intraperitoneal injection of UNC9994 (0.5 mg/kg), a β-arrestin-biased D2 receptor agonist, did not significantly affect the response of the VTA Mor-Ens and Shock-Ens to sucrose water (Figure 8F-G). However, UNC9994 attenuated the tail suspension-induced suppression of the neuronal activity in Shock-Ens but not in Mor-Ens (Figure 8H-I). The single molecule RNA fluorescence in situ hybridization (smFISH) showed that *Drd2*^+^ proportion in the VTA Mor-Ens and Shock-Ens was comparable, and the cellular fluorescent intensity of *Drd2* mRNA between the VTA Mor-Ens and Shock-Ens was similar (Figure S3). These results suggest that the differential effect of the biased agonists may not be due to the differences in the expression of *Drd2* in the ensembles.

**Figure 9:**
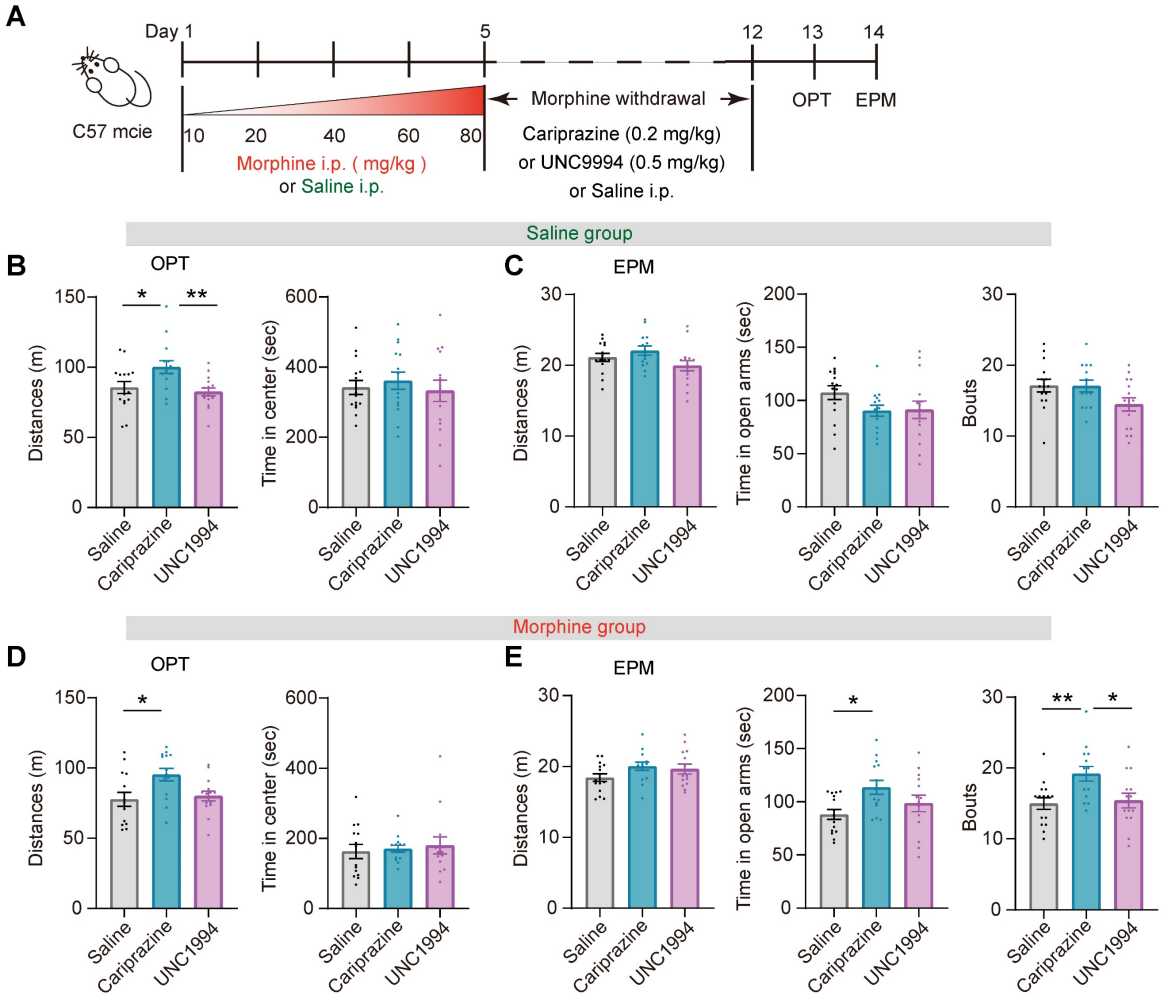
** Cariprazine alleviates the anxiety-like behaviors during morphine withdrawal. (A)** Schematic of the experimental procedure. C57BL/6 mice were intraperitoneal injected with escalating doses of morphine (10 mg/kg, 20 mg/kg, 40 mg/kg, 60 mg/kg, 80 mg/kg) or an equivalent volume of saline twice a day (9:00 a.m. and 21:00 p.m.) for 5 days. During morphine withdrawal (day 6-12), the mice were received intraperitoneal injections of cariprazine (0.2 mg/kg), UNC9994 (0.5 mg/kg) or saline. On day 13 and day 14, the open field test (OPT) and the elevated plus maze (EPM) test were conducted. **(B, C)** The 30 min OPT (B) and EPM test (C) in saline group [One-way ANOVA: (B) Saline n = 15, Cariprazine n = 14, UNC9994 n = 15. Left, F _treatment × session_ (2, 43) = 7.151, P = 0.002, Bonferroni post hoc: Saline vs Cariprazine, P = 0.015; UNC9994 vs Cariprazine P = 0.003. Right, F _treatment × session_ (2, 43) = 0.203, P = 0.817; (C) Saline n = 15, Cariprazine n = 14, UNC9994 n = 15. Left, F _treatment × session_ (2, 43) = 2.678, P = 0.081. Middle, F _treatment × session_ (2, 43) = 2.028, P = 0.145. Right, F _treatment × session_ (2, 43) = 2.997, P = 0.061]. **(D, E)** The 30 min OPT (D) and EPM test (E) in morphine group [One-way ANOVA: (D) Saline n = 14, Cariprazine n = 14, UNC9994 n = 14. Left, F _treatment × session_ (2, 41) = 4.771, P = 0.014, Bonferroni post hoc: Saline vs Cariprazine, P = 0.021; (E) Saline n = 15, Cariprazine n = 14, UNC9994 n = 14. Left, F _treatment × session_ (2, 42) = 1.925, P = 0.159. Middle, F _treatment × session_ (2, 42) = 4.029, P = 0.025, Bonferroni post hoc: Saline vs Cariprazine, P = 0.022. Right, F _treatment × session_ (2, 42) = 5.818, P = 0.006, Bonferroni post hoc: Saline vs Cariprazine, P = 0.010; Saline vs UNC9994 P = 1.000; UNC9994 vs Cariprazine P = 0.026; Kruskal-Wallis H Test: (D) Right, H = 0.457, P = 0.796]. *P < 0.05, **P < 0.01. Data are shown as mean ± SEM.

Prolonged opioid withdrawal leads to negative affective states including anxiety 45. To explore the potential function of the G-protein and β-arrestin bias signaling pathway in opioid withdrawal, mice received intraperitoneal injection of morphine at escalating doses for 5 consecutive days. The dose of each injection was 10 mg/kg on day 1, 20 mg/kg on day 2, 40 mg/kg on day 3, 60 mg/kg on day 4, and 80 mg/kg on day 5. The mice in the control group received an equivalent volume of saline injection for 5 days. From day 6 to day 12, the mice received intraperitoneal injection of cariprazine (0.2 mg/kg), UNC9994 (0.5 mg/kg) or saline twice a day. On day 13 and 14, the open field and EPM tests were conducted, respectively (Figure 9A). In the saline group, cariprazine administration resulted in an increased locomotor activity in the open field test, but UNC9994 did not have the same effect (Figure 9B). Cariprazine or UNC9994 had no significant effect on the locomotion distance, time spent in the open arms, or the frequency of entering the open arms (Bouts) in EPM test (Figure 9C). In the morphine withdrawal group, administration of cariprazine but not UNC9994 increased locomotor activity in the open field test (Figure 9D). Cariprazine also increased the time spent in the open arms and the frequency of entering the open arms in the EPM test (Figure 9E middle, 9E right), while having no significant effect on the distance moved in the EPM test (Figure 9E left). These results suggest that cariprazine may alleviate anxiety-like behaviors during morphine withdrawal.

Taken together, the study reveals the different spatial distribution, circuit connectivity and molecular characteristics of the VTA neuronal ensembles in response to various stressors, and implies potential interventions targeting different GPCR signaling pathways for opioid withdrawal or stress-related psychological disorders.

## Discussion

The present studies show that the VTA neuronal ensembles activated by morphine or foot shock have different spatial distribution and circuit connectivity. The Mor-Ens were found to be enriched in the ventral-caudal region of the VTA, whereas the Home-cage-Ens were enriched in the rostral part of the VTA (Figure 1), consistent with previous studies suggesting a regional heterogeneity within the VTA and its functional implications 39, 46, 47. These studies suggest that morphine mediates reward through dopamine-dependent effects, as evidenced by observed preference behaviors in response to optogenetic activation and the higher dopaminergic composition of the VTA Mor-Ens 17. Dopaminergic projections from the VTA to target regions, such as the nucleus accumbens, have been implicated in reward processing and reinforcement 39, 47-49. The higher proportion of dopaminergic neurons within the Mor-Ens may contribute to the observed reward-related behaviors (Figure 2).

In this study, we selected morphine i.p. and foot shock as emotional stimuli because they are high salient and relatively easy to quantify (dosage or current intensity, *etc.*) and are less affected by the internal state of mice than food or social stimuli (thirsty, hunger, *etc.*) 13, 50, 51. Research has shown that there was a significant overlap in NAc neurons responsive to both morphine and chocolate, but only a small overlap in NAc neurons responsive to morphine and restraint 16. This suggests that stimuli with different valences activate distinct neural subpopulations in NAc. Recent research showed that there was a significant overlap in VTA dopamine neurons responsive to food and social stimuli 51. This suggests that suggest these motivations with same valence (palatable rewards, social stimuli) might be encoded in a ''common currency'' in the VTA. It is yet to be explored whether the same or different VTA ensembles respond to foot shock and other aversive stimuli (such as LiCl or quinine water,* etc.*).

Brain-wide mapping of monosynaptic inputs revealed that Mor-Ens received more inputs from DMStr and MEZ, while Shock-Ens received more input from VP and LHb. The dorsal striatum (DStr) receives extensive projections from the cortex and thalamus and projects to other brain regions within the basal ganglia. It is involved in reward-related behavioral decisions 35, 52. While previous studies have focused on the projection from the striatum to the VTA, particularly the reward direct pathway mediated by medium spiny neurons (MSNs) projecting to the VTA 52. Our results indicate that there is a relatively large population of cells projecting directly from the dorsal striatum to Mor-Ens, suggesting their potential role in reward-related behaviors 53, 54. The MEZ includes the ventromedial hypothalamic nucleus (VMH) and dorsomedial nucleus of the hypothalamus (DMH). Research has shown that the ventromedial hypothalamic nucleus is involved in energy-related reward behavior, as well as the regulation of sexual behavior 36, 55, 56, stress avoidance, and defensive behavior 57, 58. The relatively strong projection from the MEZ to the VTA Mor-Ens (Figure 3) suggests the potential involvement of these ensembles in energetic reward and stress-avoidance behaviors. Shock-Ens received more input from the LHb, which is considered to play an important role in the regulation of negative emotions 38. The VP is an important node in the midbrain-limbic system, and there are two types of projections from VP to VTA: GABAergic projection and glutamatergic projection. Activation of the GABAergic projection from the VP to the VTA leads to the preference behaviors, whereas activation of the glutamatergic projection from the VP to the VTA leads to a place aversion 59. There may be a relatively high glutamatergic projection from the VP to the VTA Shock-Ens. These data suggest that the different emotional experiences, such as reward and aversion, activate different neural circuits that coverage in the VTA, and supports the notion that VTA neuronal ensembles integrate inputs from various brain regions to mediate behavioral outcomes.

Mor-Ens sends more abundant axonal arbors to the ZI and VP (Figure 4). The VP receives its major dopaminergic projections from the VTA, and dopamine in the VP has been shown to promote reward and self-administration behavior in rats 60, 61. The abundant distribution of Mor-Ens axons in the VP is consistent with the functional properties of dopaminergic neurons in the VTA-VP pathway. The ZI is considered as an important node for integrating sensory information and initiating motor behaviors. It controls of motor behavior and motivation 62. Mice with a significantly reduced in the synaptic contacts between VTA dopaminergic neurons and the ZI region exhibit depression 63. The relatively abundant distribution of Mor-Ens axons in the ZI suggests the functional properties of the VTA-ZI pathway.

The molecular profiles of the VTA Mor-Ens and Shock-Ens reveal differential gene expression patterns, particularly in neuroactive receptors and GPCR downstream signals. Neurotransmitter systems might contribute to the distinct behavioral outcomes of the VTA 6, 64. The differential expression of *Grm2*, *Gabrb1* and *Gabrg1* suggests differential glutamatergic and GABAergic signaling between Mor-Ens and Shock-Ens. Several differentially expressed genes encoding GPCRs were identified in this study. In addition, the analysis of genes transcripts involved in GPCRs downstream signaling transduction revealed 10 differentially expressed genes that play important roles in GPCR-mediated signaling cascades. Further analysis of protein-protein interaction networks analysis highlighted *Akt1*, *Plcb3*, and *Plcb1* as potential hub genes within this network. The development of biased ligands for GPCRs that act on specific downstream signaling pathways heralds an era to increase drug efficacy while reducing side effects 65. For instance, SBI-553 is a selective agonist of the neurotensin receptor 1 (NTSR1) that activates the β-arrestin signaling pathway while inhibiting the G-protein signaling pathway. This compound has been shown to reduce the side effects of NTSR1 agonists in the treatment of cocaine dependence 66. Olinvyk, a μ-receptor agonist that selectively activates the G-protein signaling pathway with minimal recruitment of β-arrestin2, has been approved by the FDA for the treatment for pain with reduced side effects, including respiratory depression and drug dependence 67.

These results provide insight into the connectivity and molecular heterogeneity of VTA neuronal ensembles in mice in response to different stressors. Understanding the integration of specific circuit and receptor signaling pathways in these ensembles may open avenues for the development of specific interventions for substance use disorders or stress-related mental disorders. In the future, the functional consequences of the cooperation between receptors and circuits in these distinct ensembles will be further investigated.

## Supplementary Material

Supplementary figures and table.

## Abbreviations

CNO: clozapine-N-oxide
CRAM: cre-dependent robust activity marking
DMStr: dorsal medial striatum
Dox: doxycycline
DEGs: differentially expressed genes
EPM: elevated plus-maze test
GPCRs: G protein-coupled receptors
IEG: immediate-early genes
KEGG: Kyoto encyclopedia of genes and genomes
LH: lateral hypothalamic area
LHb: lateral habenular nucleus
MEZ: hypothalamic medial zone
OPT: open field test
TST: tail suspension test
VP: ventral pallidum
VTA: ventral tegmental area
ZI: zona incerta
