# Supplementary material for: Morphine- and foot shock-responsive neuronal ensembles in the VTA possess different connectivity and biased GPCR signaling pathway
Source: Theranostics. 2024 Jan 12;14(3):1126–46. doi: 10.7150/thno.90792 (PMC10797299; doi:10.7150/thno.90792)
Supplement: Supplementary file 1 — Supplementary figures and table. [file thnov14p1126s1.pdf]

Figure S1 CNO significantly activates cells which expressing hM3Dq, and the number of hM3Dq$^+$ cells were comparable between the saline and foot shock groups.

(A) Mice were perfused for c-Fos staining at 90 min after CNO injection after the last behavioral experiment. **(B)** Representative images of c-Fos and hM3Dq expression in the VTA. Green: c-Fos; Red: hM3Dq; Blue: DAPI. Scale bar, left 200 μm, right 50 μm. White arrows indicate the c-Fos$^+$ hM3Dq$^+$ cells. **(C)** The proportion of c-Fos$^+$ cells in the VTA hM3Dq$^+$ cells (c-Fos$^+$ hM3Dq$^+$ cells / hM3Dq$^+$ cells). [Two-tailed Student's t-test: Saline n = 7, CNO n = 8, $t(13) = -8.119$, $P < 0.001$]. **(D)** Representative images of hM3Dq$^+$ cells in the VTA. Red: hM3Dq; Blue: DAPI. Scale bar, left 200 μm, right 50 μm. White arrows indicate the hM3Dq$^+$ cells. **(E)** The number of hM3Dq$^+$ cells per mm^2 in the VTA. [Two-tailed Student's t-test: Home-cage-Ens n = 8, Shock-Ens n = 7, $t(13) = 1.738$, $P = 0.106$]. *$P < 0.05$, **$P < 0.01$, ***$P < 0.001$. Data are shown as mean ± SEM.

Figure S2 Fluorescence signal excited by 410 nm or 470 nm laser stimulation in the fiber photometry recording.

(A) Fiber photometry recording during sucrose consumption. Blue shading indicates the period of sucrose water consumption. **(B)** Fiber photometry recording during tail suspension. Blue shading indicates the period of tail suspended. The black line represents the signal excited by 410 nm light. The green line represents the signal excited by 470 nm light.

Figure S3 Expression level of *Drd2* mRNA in the VTA Mor-Ens and Shock-Ens.

(A) Schematic of the experimental procedure for single-molecule RNA fluorescence in situ hybridization (smFISH) for *Drd2* mRNA in the VTA Mor-Ens and Shock-Ens after fiber photometry recording. **(B)** Representative images of *Drd2* mRNA signal in VTA.

Blue: DAPI; Green: *Egfp*; Red: *Drd2*. Scale bar: left 200 μm, right 25 μm. Dashed lines: outline of VTA or *Egfp*$^+$ cell. **(C)** The proportion of *Drd2*$^+$ cells in the VTA Mor-Ens or Shock-Ens (*Drd2*$^+$ *Egfp*$^+$ cells / *Egfp*$^+$ cells). [Two-tailed Student's t-test: Shock-Ens n = 5, Mor-Ens n = 5, t (8) = 1.097, P = 0.305]. **(D)** Cumulative probability curves and the violin plot depict the *Drd2* mRNA signal intensity in each *Egfp*$^+$ cell. [Kolmogorov-Smirnov test: Shock-Ens n = 2596 cells from 5 mice, Mor-Ens n = 1728 cells from 5 mice, D = 0.025, P = 0.512]. Data are shown as mean ± SEM

Fig. S1

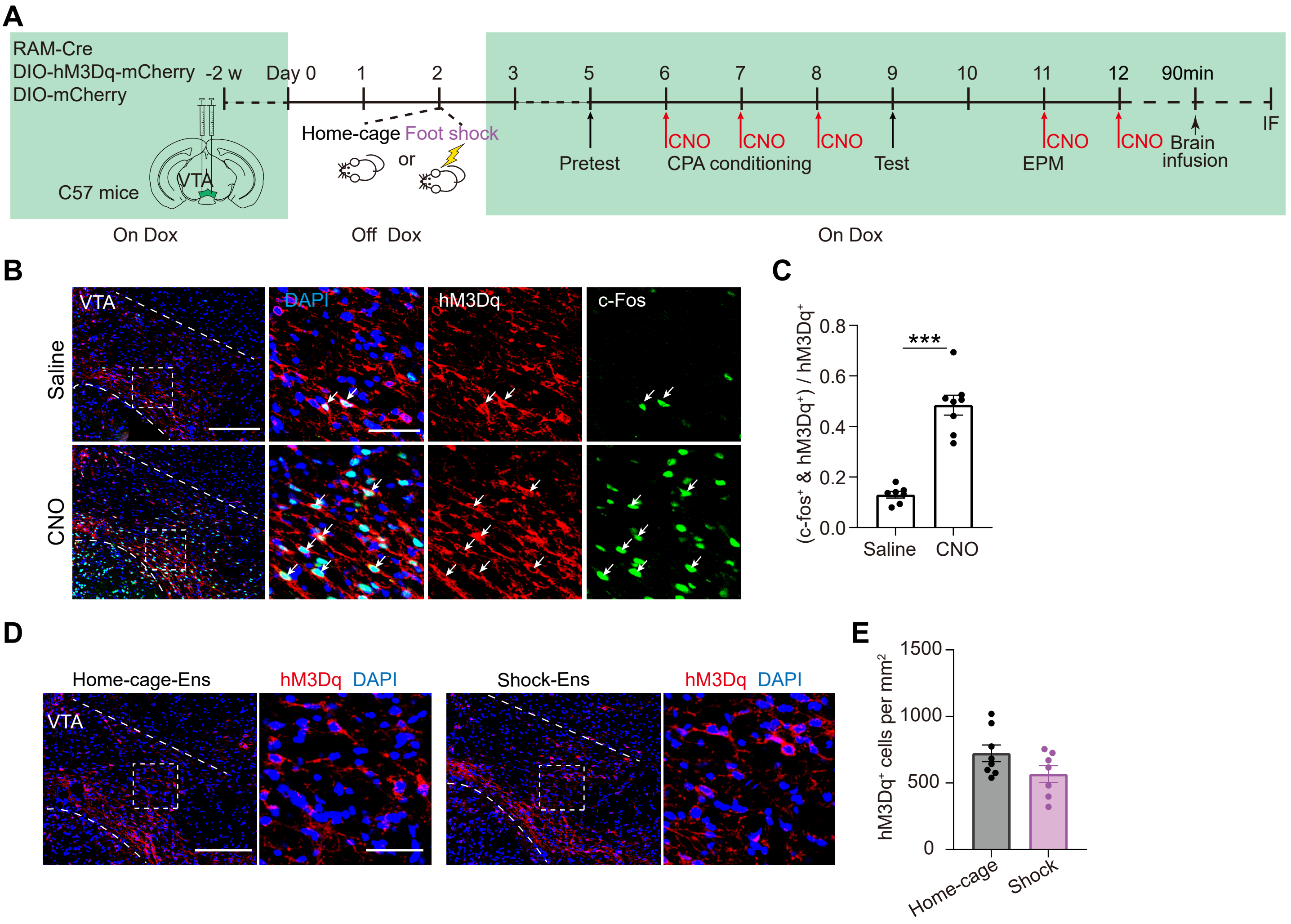

A
RAM-Cre
DIO-hM3Dq-mCherry
DIO-mCherry
-2 w
Day 0
1
2
3
5
6
7
8
9
10
11
12
90min
C57 mice
VTA
Home-cage
Foot shock
or
Pretest
CNO
CNO
CNO
CPA conditioning
Test
CNO
EPM
CNO
Brain infusion
IF
On Dox
Off Dox
On Dox
B
Saline
CNO
VTA
DAPI
hM3Dq
c-Fos
C
(c-fos+ & hM3Dq+) / hM3Dq+
0.0
0.2
0.4
0.6
0.8
***
Saline
CNO
D
Home-cage-Ens
hM3Dq DAPI
Shock-Ens
hM3Dq DAPI
VTA
E
hM3Dq+ cells per mm2
0
500
1000
1500
Home-cage
Shock

Fig. S2

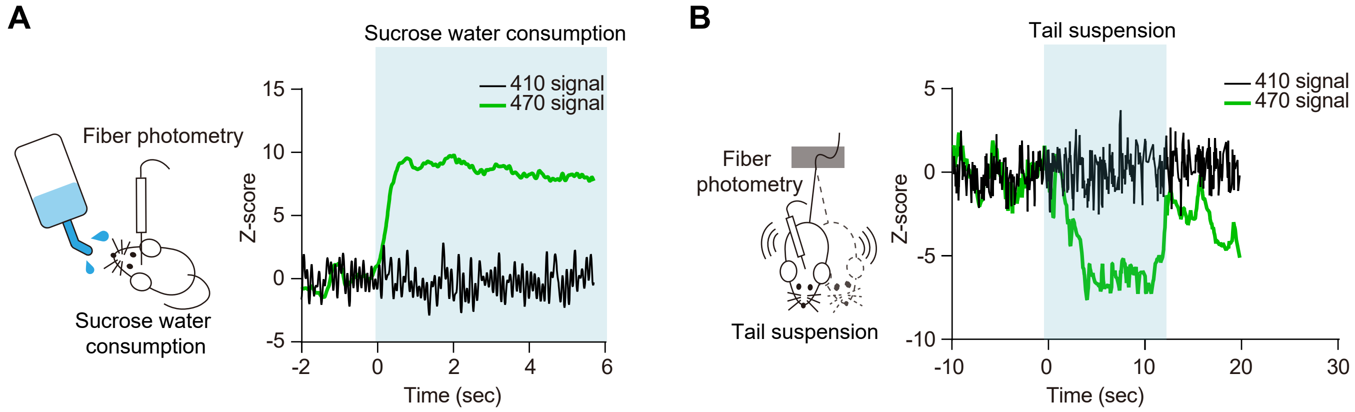
A
Fiber photometry
Sucrose water consumption
Sucrose water consumption
410 signal
470 signal
Z-score
15
10
5
0
-5
-2
0
2
4
6
Time (sec)
B
Fiber photometry
Tail suspension
Tail suspension
410 signal
470 signal
Z-score
5
0
-5
-10
-10
0
10
20
30
Time (sec)

Fig. S3

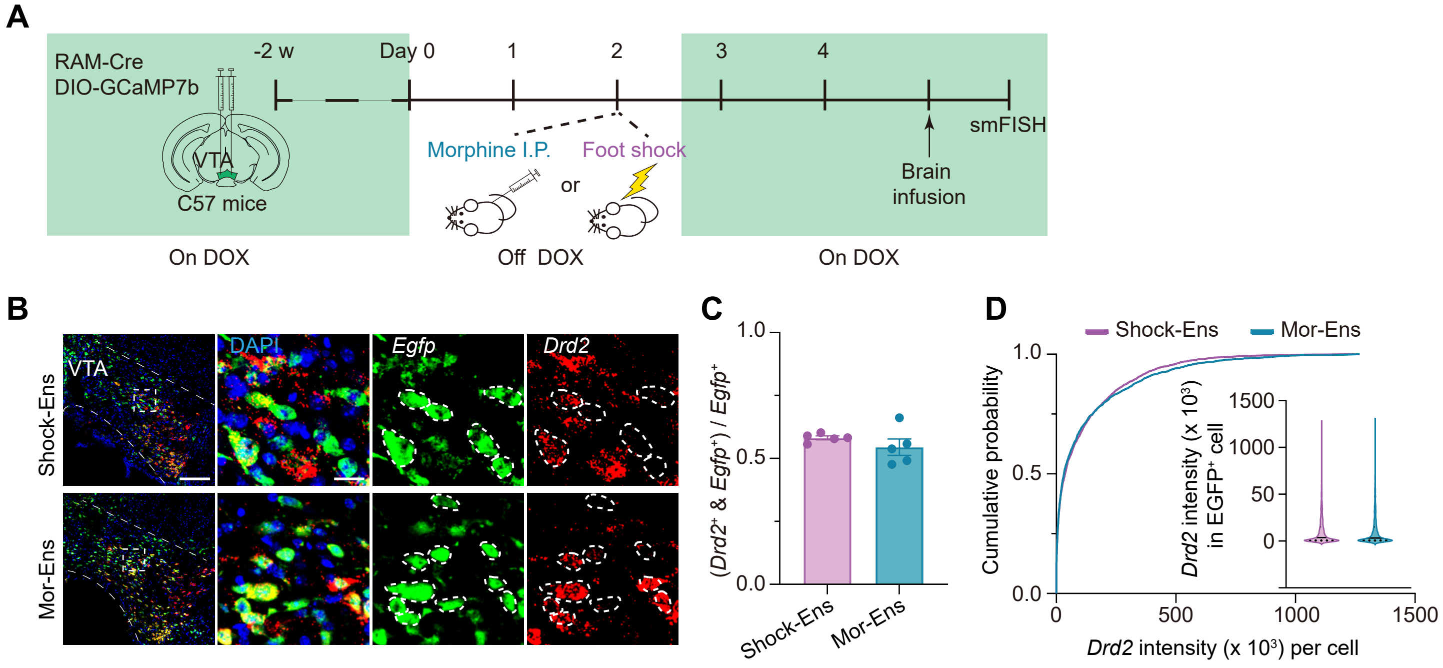

A
RAM-Cre
DIO-GCaMP7b
VTA
C57 mice
-2 w
Day 0
1
2
3
4
Morphine I.P.
or
Foot shock
Brain
infusion
smFISH
On DOX
Off DOX
On DOX
B
Shock-Ens
Mor-Ens
VTA
DAPI
Egfp
Drd2
C
(Drd2+ & Egfp+) / Egfp+
1.0
0.5
0.0
Shock-Ens
Mor-Ens
D
Shock-Ens
Mor-Ens
Cumulative probability
1.0
0.5
0.0
0
500
1000
1500
Drd2 intensity (x 10³) per cell
Drd2 intensity (x 10³)
in EGFP+ cell
1500
1000
50
0

Supplementary Table 1: Statistical detail information for figures

Figure	Response variable	groups	n define as	Normality Test (Shapiro-Wilk)	Homogeneity of variance	Statistical test	Test value	p value
1C left	c-Fos^{+} cells cell per mm^{2}	home-cage n = 7<br>shock n = 6<br>morphine n = 4	mice	df = 130, p = 0.656	Mauchly's Test of Sphericity df = 27, p = 0.102	Mixed Models	F(14, 92) = 11.28	P < 0.001
1C right	Percentage of total c-Fos^{+} cells (%)	home-cage n = 7<br>shock n = 6<br>morphine n = 4	mice	df = 130, p = 0.465	Mauchly's Test of Sphericity df = 27, p = 0.02	Mixed Models	F(14, 106) = 5.929	P < 0.001
1D	Percentage of total c-Fos^{+} cells in this section	home-cage n = 7<br>shock n = 6<br>morphine n = 4	mice	df = 51, p = 0.724	Mauchly's Test of Sphericity df = 2, p = 0.686	Two-way Repeated Measures ANOVA	F(2.744, 19.206) = 5.929	P = 0.117
1E	Percentage of total c-Fos^{+} cells in this section	home-cage n = 6<br>shock n = 6<br>morphine n = 4	mice	df = 48, p = 0.120	Mauchly's Test of Sphericity df = 2, p = 0.636	Two-way Repeated Measures ANOVA	F(2.545, 16.545) = 2.708	P = 0.086
1F	Percentage of total c-Fos^{+} cells in this section	home-cage n = 6<br>shock n = 6<br>morphine n = 4	mice	df = 48, p = 0.375	Mauchly's Test of Sphericity df = 2, p = 0.594	Two-way Repeated Measures ANOVA	F(2.377, 15.448) = 2.708	P = 0.413
1G	Percentage of total c-Fos^{+} cells in this section	home-cage n = 7<br>shock n = 6<br>morphine n = 4	mice	df = 51, p = 0.070	Mauchly's Test of Sphericity df = 2, p = 0.285	Two-way Repeated Measures ANOVA	F(4, 28) = 3.238<br>Bonferroni post hoc ml (0.6~) homecage vs morphine	P = 0.026<br>P = 0.020

Figure	Response variable	groups	n define as	Normality Test (Shapiro-Wilk)	Homogeneity of variance	Statistical test	Test value	p value
1H	Percentage of total c-Fos^{+} cells in this section	home-cage n = 5	mice	df = 45, p = 0.320	Mauchly's Test of Sphericity df = 2, p = 0.119	Two-way Repeated Measures ANOVA	F(4, 24) = 7.299	P = 0.001
		shock n = 6					Bonferroni post hoc ml (0~0.3) homecage vs morphine	P = 0.004
		morphine n = 4					Bonferroni post hoc ml (0.6~) homecage vs morphine	P = 0.001
							Bonferroni post hoc ml (0.6~) morphine vs shock	P = 0.011
1I	Percentage of total c-Fos^{+} cells in this section	home-cage n = 7	mice	df = 51, p = 0.773	Mauchly's Test of Sphericity df = 2, p = 0.471	Two-way Repeated Measures ANOVA	F(4, 28) = 9.277	P < 0.001
		shock n = 6					Bonferroni post hoc ml (0~0.3) homecage vs morphine	P < 0.001
		morphine n = 4					Bonferroni post hoc ml (0~0.3) shock vs morphine	P = 0.007
							Bonferroni post hoc ml (0.3~0.6) homecage vs morphine	P = 0.003
							Bonferroni post hoc ml (0.3~0.6) shock vs morphine	P = 0.043

Figure	Response variable	groups	n define as	Normality Test (Shapiro-Wilk)	Homogeneity of variance	Statistical test	Test value	p value
1K	distance from VTA bottom (μm)	home-cage, n = 270 cells, 7 mice	cell	df = 270, p < 0.001	——	Kruskal-Wallis H Test	H = 77.457	P < 0.001
		shock, n = 374 cells, 4 mice		df = 374, p < 0.001			Bonferroni post hoc shock vs morphine	P < 0.001
		morphine, n = 542 cells, 4 mice		df = 542, p < 0.001			Bonferroni post hoc homecage vs morphine	P < 0.001
1L	distance of paired cells (μm)	home-cage, n = 1000, 7 mice	paired distances	df = 1000, p < 0.001	——	Kruskal-Wallis H Test	H = 68.955	P < 0.001
		shock, n = 1000, 4 mice	paired distances	df = 1000, p < 0.001			Bonferroni post hoc shock vs morphine	P < 0.001
		morphine, n = 1000, 4 mice	paired distances	df = 1000, p < 0.001			Bonferroni post hoc homecage vs morphine	P < 0.001
2D left	Preference score (s)	n = 10	mice	df = 10, p = 0.081	——	Paired t-test	df = 9, t = -4.486	P = 0.002
2D right	Bouts			df = 10, p = 0.522			df = 9, t = 0.900	P = 0.392
2F left	Preference score (s)	n = 9	mice	df = 9, p = 0.483	——	Paired t-test	df = 8, t = 0.709	P = 0.498
2F right	Bouts			df = 9, p = 0.015		Wilcoxon Signed Ranks Test	Z = -0.841	P = 0.400
2H	CPP Socre (s)	Shock-Ens mCherry, n = 13	mice	df = 78, p = 0.460	Mauchly's Test of Sphericity df = 0, p = 1.000 (Greenhouse-Geisser)	Two-way Repeated Measures ANOVA (multivariate tests)	F(2, 36) = 0.916	P = 0.409
		Shock-Ens hM3Dq, n = 13						
		Homecage-Ens, hM3Dq, n = 13						

Figure	Response variable	groups	n define as	Normality Test (Shapiro-Wilk)	Homogeneity of variance	Statistical test	Test value	p value
2I	Time in open arms (s)	Shock-Ens mCherry, n = 15	mice	df = 15, p = 0.275	Levene's test F(2, 39) = 1.174, p = 0.320	One-way ANOVA	F(2, 39) = 6.022	P = 0.005
		Shock-Ens hM3Dq, n = 14		df = 14, p = 0.852			Bonferroni post hoc Shock-Ens hM3Dq vs Shock-Ens mCherry	P = 0.004
		Homecage-Ens hM3Dq, n = 13		df = 13, p = 0.183			Bonferroni post hoc Shock-Ens hM3Dq vs Homecage-Ens hM3Dq	P = 0.039
3C	$(TH^+ EGFP^+)/ EGFP^+$	Shock-Ens, n = 8	mice	df = 63, p = 0.012	Mauchly's Test of Sphericity df = 9, p = 0.250 (Greenhouse-Geisser)	Mixed Models	F (4, 39) = 3.857	P < 0.001
		Mor-Ens, n = 7					Bonferroni post hoc ml (-3.1, -3.2), Shock-Ens vs Mor-Ens	P = 0.013
							Bonferroni post hoc ml (-3.2, -3.3), Shock-Ens vs Mor-Ens	P = 0.002
							Bonferroni post hoc ml (-3.3, -3.4), Shock-Ens vs Mor-Ens	P = 0.004
3D	$(TH^+ EGFP^+)/ EGFP^+$	Shock-Ens, n = 8	mice	df = 8, p = 0.344	Levene's test F(7,6) = 0.004, p = 0.953	Two-tailed Student's t-test	df = 13, t = -4.645	P < 0.001
		Mor-Ens, n = 7		df = 7, p = 0.604				

Figure	Response variable	groups	n define as	Normality Test (Shapiro-Wilk)	Homogeneity of variance	Statistical test	Test value	p value
3F	(GABA$^+$ EGFP$^+$)/ EGFP$^+$	Shock-Ens, n = 7	mice	df = 70, p = 0.324	Mauchly's Test of Sphericity df = 9, p = 0.188	Two-way Repeated Measures ANOVA	F(4, 48) = 0.438	P = 0.780
		Mor-Ens, n = 7					Fensembles(1, 12) = 17.848	P = 0.001
							Bonferroni post hoc ml (-3.2, -3.3), Shock-Ens vs Mor-Ens	P = 0.014
							Bonferroni post hoc ml (-3.4, -3.5), Shock-Ens vs Mor-Ens	P = 0.008
							Bonferroni post hoc ml (-3.5, -3.6), Shock-Ens vs Mor-Ens	P = 0.019
3G	(GABA$^+$ EGFP$^+$)/ EGFP$^+$	Shock-Ens, n = 7	mice	df = 7, p = 0.624	Levene's test F(6,6) = 0.004, p = 0.951	Two-tailed Student's t-test	df = 12, t = 4.433	P = 0.001
		Mor-Ens, n = 7		df = 7, p = 0.747				
4C	Start neurons	Shock-Ens, n = 6	mice	df = 6, p = 0.479	Levene's test F(5,6) = 2.187, p = 0.167	Two-tailed Student's t-test	df = 11, t = 1.347	P = 0.205
		Mor-Ens, n = 7		df = 7, p = 0.052				
4D	Total input counts	Shock-Ens, n = 6	mice	df = 6, p = 0.342	Levene's test F(5,6) = 0.636, p = 0.442	Two-tailed Student's t-test	df = 11, t = -0.861	P = 0.408
		Mor-Ens, n = 7		df = 7, p = 0.720				

Figure	Response variable	groups	n define as	Normality Test (Shapiro-Wilk)	Homogeneity of variance	Statistical test	Test value	p value
4E	% Total inputs	Shock-Ens, n = 6	mice	df = 260, p = 0.268	Mauchly's Test of Sphericity df = 189, p = 0.232 (Greenhouse-Geisser)	Two-way Repeated Measures ANOVA	F(4.417, 48.592) = 2.927	P = 0.026
		Mor-Ens, n = 7					Fisher's LSD post hoc DMStr, Sock-Ens vs Mor-Ens	P = 0.014
							Fisher's LSD post hoc VP, Sock-Ens vs Mor-Ens	P = 0.002
							Fisher's LSD post hoc LHb, Sock-Ens vs Mor-Ens	P = 0.015
							Fisher's LSD post hoc MEZ, Sock-Ens vs Mor-Ens	P = 0.020
5C	Arborization on per neuron (μm^2)	Shock-Ens, n = 5	mice	VP, Shock-Ens df = 5, p = 0.417	Levene's test F(5,4) = 0.508, p = 0.499	Two-tailed Student's t-test	df = 7, t = -4.364	P = 0.003
		Mor-Ens, n = 4		VP, Mor-Ens df = 4, p = 0.270				
				ZI, Shock-Ens df = 5, p = 0.968	Levene's test F(5,4) = 2.393, p = 0.166		df = 7, t = -2.563	P = 0.037
				ZI, Mor-Ens df = 4, p = 0.429				
5D	Axon density (% VTA intensity)	Shock-Ens, n = 5	mice	ZI, Shock-Ens df = 5, p = 0.898	Levene's test F(5,4) = 0.022, p = 0.886	Two-tailed Student's t-test	df = 7, t = -2.599	P = 0.035
		Mor-Ens, n = 4		ZI, Mor-Ens df = 4, p = 0.601				

Figure	Response variable	groups	n define as	Normality Test (Shapiro-Wilk)	Homogeneity of variance	Statistical test	Test value	p value
5E	Density fraction (% all density)	Shock-Ens, n = 5	mice	Gpe, Shock-Ens df = 5, p = 0.961	Levene's test F(5,4) = 0.338, p = 0.579	Two-tailed Student's t-test	df = 7, t = -3.406	P = 0.011
		Mor-Ens, n = 4		Gpe, Mor-Ens df = 4, p = 0.466				
				ZI, Shock-Ens df = 5, p = 0.506	Levene's test F(5,4) = 0.398, p = 0.548		df = 7, t = -2.673	P = 0.032
				ZI, Mor-Ens df = 4, p = 0.797				
				LDT, Shock-Ens df = 5, p = 0.638	Levene's test F(5,4) = 2.595, p = 0.151		df = 7, t = 4.242	P = 0.004
				LDT, Mor-Ens df = 4, p = 0.579				
7C	p-AKT1 intensity per cell	Shock-Ens, n = 822 cells, 5 mice	cell	df = 822, p < 0.001	——	7C left, Mann-Whitney test	U = 240371	P < 0.001
		Mor-Ens, n = 754 cells, 5 mice		df = 754, p < 0.001		7C right, Kolmogorov-Smirnov test	D = 0.179	P < 0.001
7E	PLCβ-3 intensity per cell	Shock-Ens, n = 1340 cells, 8 mice	cell	df = 1340, p < 0.001	——	7E left, Mann-Whitney test	U = 898116	P = 0.017
		Mor-Ens, n = 1415cells, 8 mice		df = 1415, p < 0.001		7E right, Kolmogorov-Smirnov test	D = 0.056	P = 0.026
8B middle	AUC (0-5s)	n = 9	mice	df = 9, p = 0.906	——	Paired t-test	df = 8, t = -3.494	P = 0.008
8B right	Peak			df = 9, p = 0.837			df = 8, t = -2.845	P = 0.022
8C left	AUC (0-5s)	n = 8	mice	df = 8, p = 0.410	——	Paired t-test	df = 7, t = 0.061	P = 0.953
8C right	Peak			df = 8, p = 0.320			df = 7, t = -0.712	P = 0.500
8D	AUC (0-40s)	n = 9	mice	df = 9, p = 0.700	——	Paired t-test	df = 8, t = -1.565	P = 0.156
8E	AUC (0-40s)	n = 8	mice	df = 8, p = 0.937	——	Paired t-test	df = 7, t = -1.396	P = 0.205
8F middle	AUC (0-5s)	n = 12	mice	df = 12, p = 0.736	——	Paired t-test	df = 11, t = 1.876	P = 0.087
8F right	Peak			df = 12, p = 0.737			df = 11, t = 0.757	P = 0.465
8G middle	AUC (0-5s)	n = 11	mice	df = 11, p = 0.374	——	Paired t-test	df = 10, t = 1.984	P = 0.075
8G right	Peak			df = 11, p = 0.058			df = 10, t = 1.507	P = 0.163

Figure	Response variable	groups	n define as	Normality Test (Shapiro-Wilk)	Homogeneity of variance	Statistical test	Test value	p value
8H	AUC (0-40s)	n = 10	mice	df = 10, p = 0.686	——	Paired t-test	df = 9, t = 0.852	P = 0.416
8I	AUC (0-40s)	n = 8	mice	df = 8, p = 0.689	——	Paired t-test	df = 7, t = -3.714	P = 0.008
9B left	Distances (m)	Saline, n = 15	mice	df = 15, p = 0.359	F(2, 41) = 1.163, p = 0.323	One-way ANOVA	F(2, 43) = 7.151	P = 0.002
		Cariprazine, n = 14		df = 14, p = 0.105			Bonferroni post hoc Saline vs Cariprazine	P = 0.015
		UNC9994, n = 15		df = 14, p = 0.981			Bonferroni post hoc UNC9994 vs Cariprazine	P = 0.003
9B right	Time in center (s)	Saline, n = 15	mice	df = 15, p = 0.289	F(2, 41) = 1.154, p = 0.325	One-way ANOVA	F(2, 43) = 0.203	P = 0.817
		Cariprazine, n = 14		df = 14, p = 0.876				
		UNC9994, n = 15		df = 15, p = 0.980				
9C left	Distances (m)	Saline, n = 15	mice	df = 15, p = 0.590	F(2, 41) = 0.286, p = 0.753	One-way ANOVA	F(2, 43) = 2.678	P = 0.081
		Cariprazine, n = 14		df = 14, p = 0.662				
		UNC9994, n = 15		df = 15, p = 0.466				
9C middle	Time in open arms(s)	Saline, n = 15	mice	df = 15, p = 0.133	F(2, 41) = 1.248, p = 0.298	One-way ANOVA	F(2, 43) = 2.028	P = 0.145
		Cariprazine, n = 14		df = 14, p = 0.724				
		UNC9994, n = 15		df = 15, p = 0.700				
9C right	Bouts	Saline, n = 15	mice	df = 15, p = 0.528	F(2, 41) = 0.435, p = 0.650	One-way ANOVA	F(2, 43) = 2.997	P = 0.061
		Cariprazine, n = 14		df = 14, p = 0.728				
		UNC9994, n = 15		df = 15, p = 0.311				
9D left	Distances (m)	Saline, n = 14	mice	df = 14, p = 0.162	Levene's test F(2, 39) = 1.447, p = 0.248	One-way ANOVA	F(2, 41) = 4.771	P = 0.014
		Cariprazine, n = 14		df = 14, p = 0.148			Bonferroni post hoc Saline vs Cariprazine	P = 0.021
		UNC9994, n = 14		df = 14, p = 0.633				
9D right	Time in center (s)	Saline, n = 14	mice	df = 14, p = 0.232	——	Kruskal-Wallis H Test	H = 0.457	P = 0.796
		Cariprazine, n = 14		df = 14, p = 0.295				
		UNC9994, n = 14		df = 14, p = 0.004				

Figure	Response variable	groups	n define as	Normality Test (Shapiro-Wilk)	Homogeneity of variance	Statistical test	Test value	p value
9E left	Distances (m)	Saline, n = 15	mice	df = 15, p = 0.357	Levene's test F(2, 40) = 1.020, p = 0.370	One-way ANOVA	F(2, 42) = 1.925	P = 0.159
		Cariprazine, n = 14		df = 14, p = 0.753				
		UNC9994, n = 14		df = 14, p = 0.271				
9E middle	Time in open arms(s)	Saline, n = 15	mice	df = 15, p = 0.054	Levene's test F(2, 40) = 1.195, p = 0.313	One-way ANOVA	F(2, 42) = 4.029	P = 0.025
		Cariprazine, n = 14		df = 14, p = 0.306			Bonferroni post hoc Saline vs Cariprazine	P = 0.022
		UNC9994, n = 14		df = 14, p = 0.889				
9E right	Bouts	Saline, n = 15	mice	df = 15, p = 0.310	Levene's test F(2, 40) = 0.292, p = 0.749	One-way ANOVA	F(2, 42) = 5.818	P = 0.006
		Cariprazine, n = 14		df = 14, p = 0.475			Bonferroni post hoc Saline vs Cariprazine	P = 0.010
		UNC9994, n = 14		df = 14, p = 0.835			Bonferroni post hoc UNC9994 vs Cariprazine	P = 0.026
							Bonferroni post hoc Saline vs UNC9994	P = 1.000
Supplementary 1C	(c-fos$^+$ hM3Dq$^+$)/hM3Dq$^+$	Saline, n = 7	mice	df = 7, p = 0.698	Levene's test F(6,7) = 3.508, p = 0.084	Two-tailed Student's t-test	df = 13, t = -8.119	P < 0.001
		CNO, n = 8		df = 8, p = 0.447				
Supplementary 1E	hM3Dq$^+$ cells per mm^2	Home-cage, n = 8	mice	df = 8, p = 0.206	Levene's test F(7,6) = 0.00, p = 0.987	Two-tailed Student's t-test	df = 13, t = 1.738	P = 0.106
		Shock, n = 7		df = 7, p = 0.496				
Supplementary 3C	(Drd2$^+$ EGFP$^+$)/EGFP$^+$	Shock-Ens, n = 5	mice	df = 5, p = 0.873	Levene's test F(4,4) = 3.812, p = 0.087	Two-tailed Student's t-test	df = 8, t = 1.097	P = 0.305
		Mor-Ens, n = 5		df = 5, p = 0.448				
Supplementary 3D	Drd2 intensity	Shock-Ens, n = 2596 cells, 5 mice	cell	df = 2596, p < 0.001	——	Kolmogorov-Smirnov test	D = 0.025	P = 0.512
		Mor-Ens, n = 1728 cells, 5 mice		df = 1728, p < 0.001				
